# *Codonopsis tangshen* Attenuates Age-Related Pulmonary Fibrosis Through Senescence and Gut–Lung Axis Modulation

**DOI:** 10.3390/nu18193289

**Published:** 2026-10-07

**Authors:** Yi Zhao, Yumeng Gong, Han Zhu, Weide Zhang, Mingrui Cai, Changxin Yang, Olga Silva, Lingxia Xu, Yongmei Guan, Weifeng Zhu

**Affiliations:** 1 Research Center for Differentiation and Development of Basic Theory of Traditional Chinese Medicine, Jiangxi University of Chinese Medicine, Nanchang 330004, China; zhysyz2008@163.com (Y.Z.); sanyer0496@163.com (Y.G.); zhangweide@jxutcm.edu.cn (W.Z.); 18579122109@163.com (M.C.); 2 Key Laboratory of Modern Preparation of TCM, Ministry of Education, Jiangxi University of Chinese Medicine, Nanchang 330004, China; bighan314@163.com; 3 Office of International Exchange and Cooperation, Jiangxi University of Chinese Medicine, Nanchang 330004, China; 20030767@jxutcm.edu.cn; 4 Research Institute for Medicines (iMed.ULisboa), Faculty of Pharmacy, Universidade de Lisboa, Lisboa 1649-003, Portugal; odsilva@ff.ulisboa.pt; 5 Academician Workstation, Jiangxi University of Chinese Medicine, Nanchang 330004, China; xulingxia94@163.com; 6 National Key Laboratory for the Creation of Classic Prescriptions and Modern Traditional Chinese Medicine, Nanchang 330004, China

**Keywords:** *Codonopsis tangshen* extracts, aging, aging-related pulmonary fibrosis, FBXW7/TPP1 pathway, HSF1/HSP70 pathways gut microbiota, mucosal barrier function

## Abstract

Background: Cellular senescence is a critical driver of chronic inflammation, tissue remodeling, and progressive fibrosis during the aging process. *Codonopsis tangshen* Oliv. is a widely used medicinal and edible homologous plant in traditional Chinese medicine; however, its pharmacological effects on natural aging and age-related pulmonary fibrosis, as well as the underlying mechanisms, are still not fully elucidated. Methods: C57BL/6J mice were randomly divided into five groups: young control (YCtrl), natural-age control (OCtrl), high-dose CT (H-O-CT, 20.8 g/kg), low-dose CT (L-O-CT, 10.4 g/kg), and positive drug YPF (O-YPF, 20.8 g/kg), with *n* = 10 per group. A bleomycin-induced cellular senescence model in A549 cells was established in vitro to provide complementary evidence. Additionally, the composition of gut microbiota and the expression of intestinal barrier markers were analyzed to explore potential gut–lung axis involvement. Results: The results demonstrated that *C. tangshen* extract significantly decreased the histological and molecular markers of pulmonary senescence, ameliorated multiple lung function parameters, and attenuated age-related pulmonary fibrosis in aged mice. Transcriptomic analysis and protein validation further revealed that these protective effects were associated with modulation of the FBXW7/TPP1 and HSF1/HSP70 signaling pathways. Moreover, the extract reshaped the gut microbiota composition and enhanced the expression of intestinal MUC2 and tight-junction proteins, which supports possible involvement of the gut–lung axis. Conclusions: These results highlight the potential of *Codonopsis tangshen* Oliv. as a promising preclinical treatment for the intervention and management of aging and age-related fibrotic lung injury.

## 1. Introduction

Aging constitutes the primary risk factor for chronic lung diseases, which are characterized by structural and functional abnormalities such as reduced elasticity, persistent inflammation, and impaired reparative capacity [1,2]. Cellular senescence, mediated by the senescence-associated secretory phenotype (SASP), contributes to chronic inflammation, fibrosis, and various age-related pathological changes [3]. In traditional Chinese medicine (TCM), there are a total of 106 types of traditional Chinese medicinal material considered to be food–medicine homologues. These materials serve as the primary sources of nutraceuticals and functional foods [4,5]. These offer a comprehensive resource base in traditional Chinese medicine for the development of anti-aging functional products.

Basic pharmacological studies demonstrate that *C. pilosula* possesses multiple beneficial effects, including anti-pulmonary aging, immunoregulation, anti-inflammation, and anti-renal fibrosis [6,7,8]. *Codonopsis tangshen*, one of the original plants of Codonopsis Radix, is widely cultivated in northeastern Sichuan, northern Guizhou, northwestern Hunan, western Hubei, and southern Shaanxi. Its broad cultivation confers favorable varietal traits and high levels of bioactive components [9,10]. Existing studies on *C. tangshen* are limited, with only its renoprotective activity in diabetic kidney disease (DKD) models being reported [11]. Considering its presumed pharmacological similarity to *C. pilosula*, we hypothesize that *C. tangshen* may produce anti-aging activity and be therapeutically effective against age-related pulmonary fibrosis.

Aging is tightly modulated by protein ubiquitination and cellular heat stress responses, both of which are critical drivers of age-related tissue dysfunction and pulmonary pathological changes. FBXW7 is a canonical F-box protein that functions as the substrate recognition subunit of the SCF (SKP1-CUL1-F-box) E3 ubiquitin ligase complex, thereby governing the ubiquitination and degradation of multiple downstream target proteins involved in senescence and stress regulation. On the one hand, FBXW7 promotes the proteolytic degradation of telomere-binding protein TPP1 and tumor suppressor p53 [12,13]. An essential telomere protector, TPP1 maintains telomere structural stability and cellular homeostasis. FBXW7-mediated TPP1 degradation disrupts telomere capping, induces cellular senescence, and exacerbates pulmonary fibrosis. Notably, the FBXW7–TPP1 signaling axis has been verified as a key regulatory mechanism that controls type II alveolar epithelial cell (AECII) senescence in lung tissues [14]. On the other hand, FBXW7 modulates cellular stress responses and disease progression through post-translational regulation of heat shock factor 1 (HSF1) [15]. HSF1 is a core determinant of organismal longevity, and its transcriptional activity gradually declines with aging in both humans and mice. Under stress conditions, activated HSF1 translocates into the nucleus to initiate heat shock responses, while HSP70 subsequently mediates HSF1 dissociation from chromatin to restore basal homeostasis [16,17]. Functionally, HSP70 exerts potent protective effects against inflammation, tissue degeneration, and aging, and is closely involved in the progression of organ fibrosis [18,19]. Collectively, the dual regulatory effects of FBXW7 on the TPP1-mediated telomere senescence pathway and the HSF1/HSP70-dependent stress response pathway play indispensable roles in host defense against age-related pulmonary disorders, including chronic obstructive pulmonary disease [20,21].

In traditional Chinese medicine, the lung and large intestine are considered internally–externally coupled, a theoretical basis that underpins the modern-day gut–lung axis concept. Research on the gut–lung axis has confirmed that dysbiosis of the gut microbiota can aggravate the development and severity of respiratory disorders such as asthma and chronic obstructive pulmonary disease (COPD) [22,23]. Prior research has demonstrated that Lactobacillus paracasei L9 mitigates pulmonary fibrosis in aged murine models by suppressing collagen I (Col-I) synthesis through the JNK-HSF1 signaling pathway [24]. Accordingly, the gut–lung axis may serve as one critical mechanism linking intestinal inflammation to pulmonary fibrosis. On this basis, we aimed to evaluate whether *C. tangshen* is capable of retarding aging and mitigating age-associated pulmonary injury by modulating the gut microbiota.

To identify viable therapeutic strategies for age-related pulmonary fibrosis, this study investigated the protective effects of *C. tangshen* against age-related pulmonary fibrosis. It primarily examined the underlying mechanisms by which *C. tangshen* mitigates senescence and modulates the gut–lung axis through the comprehensive regulation of the FBXW7/TPP1 and HSF1/HSP70 signaling pathways.

## 2. Materials and Methods

### 2.1. Materials and Reagents

Dried *C. tangshen* was purchased from Chongqing Shennu Pharmaceutical (lot number: YC-CDS-20241018) (Chongqing, China); Yupingfeng granules were purchased from Sinopharm Group Guangdong Medi-World Pharmaceutical Co., Ltd. (lot number: 240903) (Guangzhou, China); cellular senescence β-galactosidase staining and AB-PAS staining kits were purchased from Solarbio (Beijing, China); modified Masson’s trichrome staining solution was purchased from LEAGENE (Beijing, China). The primary antibodies used in this study—beta actin, SFTPC, P21, TGF-β1, α-SMA, CDKN2A/P16-INK4A, ZO-1, occludin, claudin 3, FBXW7, STIP1, HSP70, HSF1, and MUC2—were purchased from Proteintech (Wuhan, China). Goat anti-rabbit IgG (H+L) was purchased from Elabscience (Wuhan, China); HSP90α and TPP1 were purchased from ABclonal (Wuhan, China); histone H3 (1:1000) and phospho-HSF1 (Ser303+Ser307) were purchased from Bioss (Beijing, China); PrimeScript™ RT reagent kit with gDNA eraser and TB Green^®^ Premix Ex Taq™ II were obtained from TaKaRa (Dalian, China); AST, ALT and Cre assay kits were obtained from Szybio (Wuhan, China); The Eastep™ Super Total RNA extraction kit was purchased from Promega (Madison, WI, USA); NE-PER Nuclear and Cytoplasmic Extraction Reagents were purchased from Thermo Fisher (Waltham, MA, USA);.the A549 cell line was purchased from Suzhou Starfish Biotechnology Co., Ltd. (Suhou, China); bleomycin was purchased from MCE (Monmouth Junction, NJ, USA); DMEM was purchased from Gibco (Grand Island, NY, USA); fetal bovine serum was obtained from ExCell (Suzhou, China); and an immunoprecipitation (IP/CoIP) kit was purchased from Absin (Shanghai, China).

### 2.2. Preparation of the C. tangshen Aqueous Extracts (CT)

Dried *C. tangshen* (500 g) was soaked in ten times its volume of pure water for 30 min, followed by 100 °C heating and extraction for 2 h. The mixture was then filtered through four layers of 200-mesh gauze to collect the filtrate. Subsequently, eight times the volume of water was added to the residue, which was heated and extracted for an additional 2 h. The two filtrates were combined and concentrated using a rotary evaporator, followed by freeze-drying to obtain a powder. The crude extraction yield was 44.3%.

### 2.3. Component Analysis of C. tangshen by UHPLC-Q-TOF/MS

*C. tangshen* aqueous extracts were filtered through a 0.22 μm membrane and reserved for use. A Triple TOF 5600+ high-resolution time-of-flight mass spectrometer (AB SCIEX Corporation, Marlborough, MA, USA) was utilized to obtain raw data via a Prominence Plus LC-30A ultrahigh-performance liquid chromatograph (Shimadzu Corporation, Kyoto, Japan). The analysis employed a Waters Acquity UPLC BEH C18 analytical column (2.1 mm × 100 mm, 130 Å, 1.7 µm). The mobile phase comprised water containing 0.1% (*v*/*v*) formic acid (solvent A) and acetonitrile (solvent B). The gradient elution was programmed as follows: 0.01–15 min, 5–35%B; 15–30 min, 35–95%B; 30–32 min, 95%B; 32–35 min, 95%B. The column temperature was kept at 40 °C, with a flow rate of 0.25 mL per minute. The electrospray ionization (ESI) settings were as follows: ion spray voltage floating (ISVF) at +4.0 kV and −4.5 kV; ion source temperature at 500 °C; atomizing gas pressure at 45 psi; drying gas flow rate at 10 L/min; sheath gas flow rate at 10 L/min; fragment voltage set to 140 V; and collision energy at 30 V. Mass spectrometry data were collected across a mass-to-charge ratio (*m*/*z*) range from 50 to 1250 Da in both positive and negative ion modes. Data acquisition and analysis were carried out using PeakView software 1.2. To further confirm the accuracy of key compounds, we specifically used Codonopsis glycoside I and lobetyolin as two control compounds for co-elution and fragmentation pathway comparison.

### 2.4. Effects of C. tangshen on Naturally Aging Mice

Male C57BL/6J mice aged 3 months and 18 months and weighing 25–30 g and 35–40 g, respectively, were obtained from Jiangsu Huachuang Xinnuo Pharmaceutical Technology Co., Ltd., Taizhou, China. (certificate number: SCXK (Su) 2020-0009). The animals were housed in optimal conditions at and animal experiment center (20 ± 2 °C, 12 h light/dark cycles, 40–70% humidity). All experiments were conducted in accordance with the guidelines approved by the Animal Ethics Committee of Jiangxi University of Traditional Chinese Medicine (approval JZLLSC20250527). Following 1 week of adaptive feeding, the mice were randomly assigned to five groups based on body weight (*n* = 10): 3-month-old mice (YCtrl), 18-month-old mice (OCtrl), high-dose *C. tangshen* aqueous extract group (O-H-CT, 20.8 g/kg), low-dose *C. tangshen* aqueous extract group (O-L-CT, 10.4 g/kg), and positive control group (Yupingfeng granules, O-YPF, 20.8 g/kg). According to the 2025 edition of the *Chinese Pharmacopoeia*, the recommended human dose of *C. tangshen* is 30 g. Using the body surface area conversion method, the corresponding doses for mice were calculated. Doses of 10.4 and 20.8 g/kg/day for mice correspond to 4 and 8 times the human dose, respectively. Each treatment group received oral gavage once daily for 60 days in 18-month-old mice. The YCtrl and OCtrl group was intratracheally administered 0.9% normal saline (NS) once daily for 60 days.

### 2.5. Effects of C. tangshen on Cell Senescence-Induced Cell Model

A549 cells were maintained in complete medium at 37 °C with 5% CO_2_. Initially, the appropriate concentration of *C. tangshen* aqueous extract for treatment of A549 cells was evaluated using the CCK8 assay. To determine the inhibitory concentration, cells were seeded in 96-well plates at a density of 3.0 × 10^3^ cells per well and exposed to a series of CT concentrations (0, 2, 4, 8, 16, and 32 mg/mL). After 48 h of incubation, 10 µL of CCK8 reagent was added to each well, followed by a 4 h incubation period in the dark at 37 °C. Absorbance was subsequently measured at 450 nm using a microplate reader.

To develop the cellular senescence model, cells were treated with varying concentrations of bleomycin (5 µg/mL, 10 µg/mL, 20 µg/mL, 30 µg/mL, and 40 µg/mL) for 24 h to induce senescence. Subsequently, senescent cells were identified using SA-β-gal staining. Following this, cells were exposed to CT at concentrations of 4, 8, and 16 mg/mL for 24 h, after which RNA was extracted to evaluate the effect of CT on ameliorating cellular senescence.

### 2.6. Pulmonary Ventilation Function Test and AST, ALT, and CRE Levels

Following anesthesia administration with 1% sodium pentobarbital, tracheal intubation was conducted. Pulmonary function parameters, including peak inspiratory flow (PIF), peak expiratory flow (PEF), tidal volume (TV), minute ventilation (MV), bronchoconstriction index (Penh), and dynamic lung compliance (Cdyn), were assessed utilizing a Small Animal Lung Function Testing System (EMKA EMKA-WBP, Paris, France). Comparative analyses of pulmonary ventilation function across the experimental groups were performed. Additionally, serum levels of aspartate aminotransferase (AST), alanine aminotransferase (ALT), and creatinine (CRE) were quantified employing a fully automated biochemical analyzer in accordance with the manufacturer’s reagent kit protocols.

### 2.7. Hematoxylin-and-Eosin Staining, Masson Staining

Segments of the pulmonary tissue samples were fixed in 4% paraformaldehyde, followed by embedding in paraffin. The paraffin-embedded sections were subsequently stained with hematoxylin and eosin (H&E) and Masson’s trichrome to evaluate lung tissue damage related to inflammation and fibrosis among the various experimental groups. Furthermore, Masson’s trichrome staining was employed to identify cardiac fibrosis within the tissue sections. Finally, the images were examined and analyzed using ImageJ software 1.54.

### 2.8. Senescence-Associated Beta-Galactosidase (SA-Beta-Gal) Staining

Following the instructions provided with the SA-β-gal staining kit, the cell and tissue samples were rinsed with phosphate-buffered saline (PBS), fixed, and then incubated overnight at 37 °C in the absence of CO_2_ with the SA-β-gal staining solution. Following incubation, the samples were washed with PBS. The reaction was subsequently terminated using 70% ethanol, followed by counterstaining with eosin. Standard procedures for dehydration, clearing, and mounting were then performed. The presence of blue staining, indicative of SA-β-gal activity, was examined under a light microscope to assess the degree of cellular senescence in lung tissue. Images were acquired using an optical microscope, and the proportion of SA-β-gal positive cells was quantitatively analyzed.

### 2.9. RNA Sequencing and Ingenuity Pathway Analysis

Every three samples of lung tissue from YCtrl, OCtrl, O-H-CT groups, and tissue were extracted using total RNA extraction kit. The integrity of the RNA samples was assessed with the Agilent Bioanalyzer 2100 system (Agilent Technologies, Santa Clara, CA, USA), yielding RIN ≥ 7.0. RNA sequencing analysis was performed on the DNBSEQ platform (BGI, Shenzhen, China). The processed data were aligned to the reference genome utilizing HISAT2 (version 2.2.1) [25] and to the reference gene set employing Bowtie2 (version 2.4.5) [26]. Gene expression quantification was carried out using RSEM (version 1.3.1) [27]. Differential gene expression analysis was performed with DESeq2 (version 1.34.0) [28], genes satisfying the criteria of *p* < 0.05 and |log fold change| > 1.5 were selected for differential expression analysis. The differentially expressed genes (DEGs) identified were submitted to Ingenuity Pathway Analysis (IPA) software (Spring Release 2025) for both core and upstream analyses. The enrichment of focus genes within IPA networks was assessed using Fisher’s exact test. Additionally, IPA determined the principal functions associated with each network by calculating enrichment scores (z-scores), thereby emphasizing the predicted biological relevance of the results.

### 2.10. Immunofluorescence (IF)

Immunofluorescence staining was employed to assess the expression of SP-C, P21, TGF-β1, and α-SMA in lung tissue samples. Paraffin-embedded sections were first deparaffinized and subjected to antigen retrieval using EDTA buffer via microwave treatment. The sections were then blocked with 10% goat serum at room temperature for one hour. Subsequently, the sections were incubated overnight at 4 °C with the respective primary antibodies (SP-C, P21, TGF-β1, and α-SMA), each diluted at 1:200. Following PBS washes, species-specific fluorescent secondary antibodies conjugated to Alexa Fluor^®^ 488 or 594 (diluted 1:300) were applied and incubated in the dark at room temperature for 1.5 h. After additional PBS washes, nuclear staining was performed using DAPI for 10 min. The sections were then mounted with an antifade medium and examined under a fluorescence microscope. Quantitative analysis of the average fluorescence intensity for each protein within the lung tissue was conducted using ImageJ software.

### 2.11. Western Blot Analysis

Lung tissue samples were homogenized in ice-cold RIPA buffer supplemented with phenylmethylsulfonyl fluoride. Protein concentrations were determined using a BCA protein assay kit. The samples were then subjected to SDS-PAGE electrophoresis and subsequently transferred onto PVDF membranes. Following a blocking step with 5% skimmed milk at room temperature for one hour, the membranes were incubated overnight at 4 °C with primary antibodies diluted at 1:1000. After washing, the membranes were incubated with the corresponding secondary antibodies for one hour and developed using enhanced chemiluminescence (ECL) reagents. Densitometric analysis of the resulting blots was performed using ImageJ software.

### 2.12. RT-qPCR

Total RNA was extracted and then reverse-transcribed into cDNA using commercial kits (TaKaRa) according to the manufacturer’s instructions. RT-qPCR was performed using SYBR Green Master Mix (TaKaRa) with the following thermocycling program: 95 °C for 2 min, then 95 °C for 5 s, 60 °C for 10 s, and 72 °C for 15 s for 40 cycles. Relative gene expression was determined using the 2^−ΔΔCq^ method. mRNA levels were normalized to β-actin. The primer sequences are provided in Supplementary Table S1.

### 2.13. Analysis of Gut Microbiota

The 16S rRNA sequencing analysis of the cecal microbiota was conducted using the BGI platform (BGI, Shenzhen, China). Initially, genomic DNA was extracted from cecal contents. The V3–V4 region of the 16S rRNA gene was amplified via PCR employing the forward primer 338F (5′-ACTCCTACGGGAGGCAGCAG-3′) and the reverse primer 806R (5′-GGACTACHVGGGTWTCTAAT-3′). Subsequently, the PCR products were purified and sequenced on the Illumina NextSeq 2000 platform (Illumina, San Diego, CA, USA). Following sequencing, alpha diversity was assessed using diversity indices, including Shannon and Simpson, as well as richness estimators ACE and Chao1, through QIIME software. Beta diversity was evaluated by principal component analysis (PCA), partial least squares discriminant analysis (PLS-DA), and permutational ANOVA (PERMANOVA) utilizing R software version 3.1.1. Species abundance analyses were performed using Metastats software. We applied the Benjamini–Hochberg (BH) false-discovery rate (FDR) procedure to the raw *p*-values from the differential abundance analysis. Taxa with an adjusted *p*-value < 0.05 were considered statistically significant.

### 2.14. CO-IP Analysis

Cellular senescent fractions were isolated as previously described. For immunoprecipitation, 500 µg of total protein was incubated with equimolar amounts of either anti-FBXW7 or anti-HSF1 antibodies, under continuous gentle rotation at 4 °C overnight. The resulting immunocomplexes were then captured by adding 40 µL of protein A+G agarose beads (Beyotime Institute of Biotechnology, Shanghai, China), followed by gentle rotation at 4 °C for an additional 4 h. Subsequently, the mixture was centrifuged at 1000× *g* for 5 min at 4 °C, and the supernatant was carefully discarded. The immunoprecipitated pellet was washed five times with ice-cold phosphate-buffered saline (PBS). After washing, the pellet was resuspended in sodium dodecyl sulfate (SDS) sample buffer and heated at 100 °C for 5 min to elute the bound proteins from the agarose beads. The eluted supernatant containing the dissociated immunocomplexes was collected by brief centrifugation and prepared for subsequent analyses.

### 2.15. Molecular Docking

Molecular docking between the active components of CT and the core target HSF1 was conducted. The three-dimensional structure of HSF1 (5D5V, pdb 00005d5v) was retrieved from the RCSB PDB (https://www.rcsb.org/). Water molecules and original ligands were removed from the protein structure using PyMol-2.6.0a0. The chemical structures of the active compounds, provided in Mol2 format, were retrieved from the TCMSP database. Subsequently, hydrogen atoms were added, and the macromolecular structures were prepared using AutoDock 4.2.6. Molecular docking simulations were then performed. The docking pose exhibiting the lowest binding energy was selected as the most favorable binding model, and the results were visualized with PyMol.

### 2.16. Statistical Analysis

Data were visualized using GraphPad Prism version 8.0. Quantitative analysis of the average fluorescence intensity for each protein within lung tissue was performed using ImageJ software. Grayscale values from Western blot results were analyzed with Image Lab software. Spearman correlation coefficients were calculated using SPSS Statistics version 26. Data are presented as means ± standard deviation (SD). One-way analysis of variance (ANOVA), followed by Duncan’s multiple range test, was conducted using SPSS Statistics version 26 (IBM, Armonk, NY, USA) to assess statistical significance. A *p*-value less than 0.05 was considered statistically significant.

## 3. Results

### 3.1. UPLC-Q-TOF-MS/MS Analysis of CT

The total ion chromatograms of *C. tangshen* provided insights into the chemical composition of the constituent compounds (Figure 1A,B). UPLC-Q-TOF-MS/MS profiling of the *C. tangshen* extract tentatively characterized 66 constituents, including alkaloids/nitrogen-containing compounds, phenolic and organic acids, phenylpropanoids, terpenes, flavonoids, alkynes, and other minor constituents. A detailed annotation list is provided in Table 1. Codonopsis glycoside I and lobetyolin were used as control compounds for co-elution and fragmentation pathway comparison (Figure 1C). First, high-resolution mass spectrometry revealed a parent ion peak [M-H]^−^ at *m*/*z* 677.2253 in negative ion mode. Based on the elemental composition, the molecular formula was determined to be C_29_H_42_O_18_ (mass error < 5 ppm). The MS^2^ spectrum showed that the parent ion [M-H]^−^ at *m*/*z* 677.2230 fragmented to produce characteristic ions at *m*/*z* 161.0463 (glucose residue) and *m*/*z* 497.1644, which further lost one molecule of COOH to form the fragment at *m*/*z* 453.1741. The retention time and all fragmentation pathways of this compound were completely consistent with those of the reference compound Codonopsis glycoside I, confirming its accurate identification. Second, a [M-H]^−^ ion peak at *m*/*z* 395.1688, corresponding to the molecular formula C_20_H_28_O_8_. Its MS^2^ spectrum revealed that the parent ion lost a glucose moiety, producing the characteristic fragment [M-C_6_H_11_O_5_]^−^ at *m*/*z* 215.1084 and another fragment at *m*/*z* 179.057. This fragment further lost a -COOH group, resulting in a fragment at *m*/*z* 143.0735. Comparison with the retention time and fragmentation pattern of the lobetyolin reference standard, then confirm that it is this component in Table 2.

### 3.2. Effects of C. tangshen on Body Weight, Histological Examination, and Liver and Kidney Function

All groups exhibited a progressive increase in body weight during the administration phase. Notably, the rate of weight gain in the OCtrl group was lower than that observed in the YCtrl group. In contrast, the treatment groups demonstrated significantly higher weight gain rates compared to the OCtrl group, indicating that CT administration promotes body weight gain in aged mice (Figure 2A,B).

Histological analysis via hematoxylin and eosin (HE) staining (Figure 2D), coupled with quantification of the edema index (Figure 2C), demonstrated a significant elevation in perivascular edema in the lung tissue of the OCtrl group compared to the YCtrl group (*p* < 0.01). Administration of H-O-CT significantly decreased the perivascular edema index relative to the OCtrl group (*p* < 0.01), whereas the L-O-CT group exhibited a reduction that did not reach statistical significance.

In the end, the impact of CT drugs on hepatic and renal function was assessed through the measurement of AST, ALT, and CRE levels. The findings demonstrated no significant variations in AST and ALT levels across the groups. However, a slight increase in CRE levels was observed in the OCtrl group. Subsequent to CT administration, a reduction in CRE levels was noted (*p* < 0.05) (Figure 2E).

### 3.3. Aging-Associated SA-β-gal Staining and IF Analysis of SP-C and P21

The results of SA-β-gal staining reveal an increased number of senescent cells within the lung tissue of the OCtrl group relative to the YCtrl group. Treatment with both high and low doses of CT reduced senescent cell counts compared to the OCtrl group, suggesting that CT effectively mitigates lung tissue aging in aged mice (Figure 3A). In comparison to the YCtrl group, the OCtrl group exhibited a significant reduction in SP-C-positive expression within mouse lung tissue (*p* < 0.01), alongside notable increases in P21 (*p* < 0.01) levels. Relative to the OCtrl group, lung tissues from mice treated with CT demonstrated a significant elevation in SP-C-positive expression (*p* < 0.05, *p* < 0.01), as well as significant decreases in P21 positive expression (*p* < 0.01). These findings indicate that CT may enhance the population of alveolar type II epithelial cells and ameliorate lung tissue aging in aged mice (Figure 3B–E).

### 3.4. Pulmonary Ventilation Parameters, Masson Staining, and IF Analysis of α-SMA and TGF-β1 in Aged Mice

Compared to the YCtrl group, mice in the OCtrl group exhibited significant reductions in PIF (*p* < 0.01), accompanied by modest decreases in Cdyn, TV, MV, and PEF, as well as a pronounced increase in enhanced Penh (*p* < 0.01). Administration of CT significantly increased TV, MV, PIF, and PEF, showed a tendency to improve Cdyn, and reduced Penh (*p* < 0.05) relative to the OCtrl group (Figure 4A). These results suggest that CT has a beneficial effect on pulmonary function in aged mice.

The assessment of pulmonary fibrosis using Masson’s trichrome staining revealed a significant increase in lung tissue fibrosis in the OCtrl group compared to the YCtrl group (*p* < 0.01). Administration of both doses of CT significantly attenuated the degree of lung fibrosis relative to the OCtrl mice (Figure 4B,E). Furthermore, immunofluorescence analysis demonstrated a marked elevation in TGF-β1 and α-SMA positive expression in lung tissue of the OCtrl group compared to the YCtrl group (*p* < 0.01). Treatment with CT significantly reduced TGF-β1 and α-SMA expression relative to the OCtrl mice (*p* < 0.01) (Figure 4C,D,F,G). Collectively, these findings suggest that CT mitigates age-related pulmonary fibrosis, which is associated with the downregulation of α-SMA and TGF-β1 expression.

### 3.5. Differential Gene Expression and Ingenuity Pathway Analysis (IPA) Analysis

Genes satisfying the criteria of *p* < 0.05 and |log fold change| > 1.5 were selected for differential expression analysis. The OCtrl group exhibited 39 upregulated and 79 downregulated differentially expressed genes (DEGs) relative to the YCtrl group (Figure 5A). After treatment with CT, there are 127 genes upregulated and 130 genes downregulated compared to the OCtrl group (Figure 5B). These DEGs were analyzed using IPA network analysis to elucidate interaction networks. reveal interactions among the aging-associated molecule TP53, the inflammatory cytokine IL-1β, and the pro-fibrotic factor TGF-β1 within the lung tissue of the aged control group, suggesting a potential mechanistic link between cellular senescence and fibrosis progression. Additionally, interactions involving NF-κB, IL-22, and AKT were identified in the OCtrl group (Figure 5C,D).

Canonical pathway analysis conducted using IPA software identified three pathways with Z scores ≥ 2 (Figure 5F). These pathways included the HSP90 chaperone cycle for steroid hormone receptors in the presence of ligand, the protein ubiquitination pathway, and the cellular response to heat stress. Additionally, upstream regulator analysis predicted potential upstream molecules responsible for the observed gene expression changes, encompassing microRNAs and transcription factors. Notably, FBXW7 was identified as a key upstream regulator associated with CT treatment, suggesting the need for further investigation into the protein ubiquitination mechanisms involving FBXW7 (Figure 5E).

### 3.6. Effects of C. tangshen on Fibrosis and SASP Markers and Pulmonary Tight-Junction Protein Expression in Aging Mice

First, there was gene expression on fibrosis and SASP markers. Compared with the YCtrl group, the OCtrl group showed an upward trend in gene expression levels of TGF-β1 and α-SMA (*p* < 0.05, *p* < 0.01). Following CT treatment, there was a noticeable decrease in the levels of TGF-β1 and α-SMA expression to varying extents (*p* < 0.05, *p* < 0.01) (Figure 6A). The SASP-related genes, such as P21, CXCL1, and CXCL2, exhibited an increasing trend in the OCtrl mice (*p* < 0.01). Treated with CT, there was a significant decrease in the expression levels of P21, CXCL1, and CXCL2 (*p* < 0.01) (Figure 6B). Furthermore, the fibrosis protein expression of TGF-β1, α-SMA, and senescence-associated proteins P53 and P21 had the same efficacy. The OCtrl group showed higher protein levels of fibrosis markers α-SMA and TGF-β1 (*p* < 0.05, *p* < 0.01), along with increased levels of senescence-related proteins P53 and P21 (*p* < 0.05, *p* < 0.01). After treatment with CT, the levels of α-SMA, TGF-β1, P53, and P21 proteins were significantly decreased, with statistical significance (Figure 6C,D). These results suggest that CT effectively alleviates lung aging and pulmonary fibrosis.

To better understand how CT affects the pulmonary mucosal barrier, we analyzed the levels of TLR4, ZO-1, occludin, and claudin 3 proteins. The expression of TLR4 protein was significantly elevated in the O-Ctrl group (*p* < 0.05), whereas the levels of ZO-1, occludin, and claudin 3 were significantly reduced (*p* < 0.05, *p* < 0.01). Following treatment with CT, TLR4 expression significantly decreased, while the levels of ZO-1, occludin, and claudin 3 were significantly increased (Figure 6E,F). These findings suggest that CT enhances the integrity of the pulmonary mucosal barrier in aged mice.

### 3.7. C. tangshen Effects on FBXW7/TPP1 and HSF1/HSP70 Pathway in Aged Pulmonary Tissue

The RT-qPCR analysis results of heat shock-related genes in aged pulmonary tissue showed that compared to the YCtrl group, the OCtrl group had a significant increase in the expression of HSP70-1, HSP70-2, HSPB1, HSPH1, STIP1 and DNAJA1 (*p* < 0.05, *p* < 0.01), while HSF1 expression was significantly decreased. Treatment with CT led to a notable decrease in the expression levels of HSP70-1, HSP70-2, HSPA1B, HSPB1, HSPH1, STIP1, and DNAJA1 (*p* < 0.05, *p* < 0.01), along with an increase in HSF1 expression (Figure 7A).

HSF1 also harbors a CPD motif and phosphorylation at Ser303 and Ser307 is necessary for its binding by FBXW7 [29]. The aging pulmonary tissue showed a notable decrease in nuclear HSF1 protein levels (*p* < 0.01), a significant rise in cytoplasmic HSF1 protein levels (*p* < 0.05), and a marked reduction in p-HSF1 (Ser303 + Ser307) expression (*p* < 0.01). Moreover, relative to the OCtrl group, treatment with CT significantly increased nuclear HSF1 protein expression (*p* < 0.01), decreased cytoplasmic HSF1 protein expression, and elevated p-HSF1 expression (*p* < 0.01) (Figure 7B,C). Additionally, the OCtrl group showed significantly lower protein levels of FBXW7, STIP1, HSP70, and TPP1 compared to the YCtrl group (*p* < 0.05). After CT treatment, the protein expression levels of FBXW7, STIP1, TPP1, and HSP70 were significantly increased compared to the OCtrl group (Figure 7D,E). Collectively, these findings suggest that CT enhances the activity of HSF1, and that the FBXW7/HSF1/HSP70 pathway contributes to the attenuation of aging-related fibrosis.

### 3.8. Effects of C. tangshen on Intestinal Barrier Function and Related Proteins in Aged Colon Tissue

HE staining revealed that the colon tissue of OCtrl mice exhibited detachment of the basement membrane. In contrast, mice treated with varying doses of CT demonstrated an improvement in basement membrane integrity compared to the OCtrl group (Figure 8A). AB-PAS staining revealed that the YCtrl group had a healthy colonic mucosa characterized by closely packed crypts, numerous goblet cells, and thick blue-purple acidic mucus, with no signs of mucosal damage or inflammation. In contrast, the OCtrl group showed damaged mucosa, loosely arranged crypts, a reduced number of goblet cells, and weak mucus staining, suggesting mucosal injury and decreased mucus production. Treatment with CT partially restored the damaged mucosa to its intact state, increased the number of goblet cells, and enhanced the intestinal mucosal barrier (Figure 8B). IF staining of MUC2 showed that the OCtrl group had a significantly lower fluorescence intensity (*p* < 0.01) and a marked reduction in the number of goblet cells. Treatment with CT significantly increased MUC2 expression (*p* < 0.01) (Figure 8C,D). Furthermore, the expression levels of proteins associated with the integrity of the colon tissue barrier were measured. The OCtrl group exhibited reduced levels of ZO-1, occludin, claudin 3, and claudin 1 proteins (*p* < 0.01). Following CT treatment, the expression of these proteins was significantly elevated compared to the OCtrl group (*p* < 0.05, *p* < 0.01) (Figure 8E,F). These findings demonstrate that CT provides protective effects against basement membrane damage, effectively restores MUC2 expression, and enhances colon barrier integrity.

### 3.9. Effects of C. tangshen on Gut Microbiota

For α-diversity, the OCtrl mice exhibited lower values in the Ace, Chao, and Sobs indices compared to the YCtrl mice, indicating a reduction in species richness. Conversely, treatment with CT showed increased values in these indices, suggesting an enhancement in species richness (Figure 9A). β-diversity analysis revealed no significant differences between the YCtrl and OCtrl groups, CT treatment enhance its diversity (Figure 9B). Non-metric multidimensional scaling (NMDS) and principal coordinate analysis (PCoA) revealed greater spatial dispersion in the OCtrl group compared to the other four groups, indicating a distinctly different microbial community structure. PCoA, supplemented by database comparisons, was utilized to evaluate differences in microbial abundance at the family level (Figure 9C,D).

At the family level, the ten most predominant gut microbiota identified were Lachnospiraceae, Oscillospiraceae, Rikenellaceae, Muribaculaceae, Prevotellaceae, Akkermansiaceae, Anaeroplasmataceae, Bacteroidaceae, Odoribacteraceae, and Lactobacillaceae (Figure 9E). The data indicated that OCtrl mice exhibited increased abundance of Lactobacillaceae and decreased abundance of Akkermansiaceae and Oscillospiraceae compared to YCtrl mice. Conversely, the O-L-CT treatment showed reduced abundance of Lactobacillaceae alongside concomitant increases in Oscillospiraceae, with notably elevated abundance of Akkermansiaceae and Bacteroidaceae (*p* < 0.01, *p* < 0.05). No significant differences were observed in the abundance of Lachnospiraceae among the groups (Figure 9F).

At the genus level, the ten most predominant gut microbiota were Alistipes, Kineothrix, Flintibacter, Akkermansia, Acetatifactor, Vescimonas, Sporofaciens, Duncaniella, Lawsonibacter, and Muribaculum (Figure 9G). The OCtrl mice exhibited increased levels of Acetatifactor and decreased abundance of Kineothrix, Flintibacter, Akkermansia, Vescimonas, and Lawsonibacter compared to the YCtrl group. Treatment with O-L-CT resulted in a reduction of Acetatifactor and elevated levels of Kineothrix, Vescimonas, and Lawsonibacter. Notably, there was a statistically significant increase in the abundance of Flintibacter and Akkermansia (*p* < 0.05, *p* < 0.01) (Figure 9H).

### 3.10. Co-IP Analysis and Effects of C. tangshen on Cell Senescence and Forosis Model

A549 cells were treated with CT at concentrations of 0, 2, 4, 8, 16, and 32 mg/mL for 24 and 48 h, with no significant inhibition of cell growth observed. Consequently, concentrations of 2, 4, and 8 mg/mL were selected for subsequent drug treatments (Figure 10A). Further evaluation of bleomycin-induced cellular senescence models across various concentrations revealed no significant dose-dependent effects based on SA-β-gal staining, leading to the selection of 5 mg/mL as the modeling concentration for future experiments (Figure 10B). Finally, RT-qPCR was conducted to examine the effects of CT on the expression of genes including P53, P21, P16, TPP1, FBXW7, HSF1, MMP3, MMP9, and TGF-β1 within the senescence model. The results demonstrated significant upregulation of senescence-associated genes P53, P21, P16, MMP3 and MMP9 (*p* < 0.05 or *p* < 0.01), as well as increased expression of fibrosis-related genes FBXW7 and TGF-β1 (all *p* < 0.01), alongside a decrease in TPP1 and HSF1 expression (*p* < 0.05, *p* < 0.01). Treatment with CT significantly reduced the expression of senescence-related genes and decreased levels of FBXW7 and TGF-β1, whereas its effect on HSF1 expression was not statistically significant (Figure 10C).

Finally, Co-IP was used to confirm the interaction between FBXW7 and HSF1. Immunoprecipitation was conducted utilizing anti-HSF1 and anti-FBXW7 antibodies, respectively, followed by Western blot analysis to detect FBXW7 and HSF1 proteins (Figure 10D,E). The findings demonstrated the presence of both FBXW7 and HSF1 proteins, indicating an interaction between these two proteins, which was further augmented upon BLM induction.

### 3.11. Molecular Docking Verification of Core Targets

HSF1 is made up of an N-terminal DNA-binding domain (DBD), a coiled-coil oligomerization domain, a regulatory domain, and a transactivation domain. When cells are under stress, HSF1 forms trimers through its coiled-coil domain and binds to the promoters of heat shock protein genes such as HSP70 [30]. Molecular docking was conducted between 66 components of CT and the key target HSF1. The active compounds identified were stigmasterol 3-O-beta-D-glucopyranoside, fritillaziebinol, atractylenolide III, tanshilactone, glycitein and coelogin, with binding energies of −6.763, −6.043, −6.034, −5.799, −5.383, and −5.144 kcal/mol, respectively (Figure 11). Since all binding energies were below −5 kcal/mol, these results suggest strong binding affinity, high biological interaction, and stable molecular conformations between the main components and the target protein HSF1. The current findings initially pinpoint active ingredients in CT that interact with HSF1, offering a basis for further research into the material composition of CT.

## 4. Discussion

China serves as the primary distribution center and main production area for the genus Codonopsis, encompassing approximately 39 species, worldwide. Among these, *C. pilosula* (CP), *C. pilosula* var. modesta (CPM) and *C. tangshen* (CT) are included in the 2025 edition of the *Pharmacopoeia*. However, these species exhibit significant differences in their phytochemical compositions. Liquid chromatography–mass spectrometry (LC-MS) analyses have demonstrated distinct small-molecule metabolite profiles for each species, identifying 3, 9, and 11 potential biomarkers, respectively [30]. Current research predominantly focuses on *C. pilosula*, which has been shown to exert protective effects on aging lung tissue, improve pulmonary function, and reduce airway inflammation [7]. In our data, codonopsis glycoside I and lobetyolin were identified, which was consistent with literature reports [31,32]. Lobetyolin modulates Nrf2/NF-κB-mediated inflammation and oxidative stress [33]. Lobetyolin decreases the expression of TNF-alpha and HIF-1 alpha, decreases CD16/CD32 levels and increases CD206 levels, which shows that lobetyolin can protect BV2 cells from OGD/R-induced damage by regulating the phenotypic polarization of BV2 and decreasing inflammatory responses [34]. Nonetheless, no studies to date have investigated the effects of *C. tangshen* on delayed aging or the amelioration of lung damage. Yupingfengsan (YPF) has been utilized for centuries in China and Southeast Asia to treat a range of illnesses, with evidence showing that YPF possesses immune-regulating and antiviral effects [35,36,37]. Clinically, YPF is used to treat chronic obstructive pulmonary disease (COPD) and to prevent COVID-19 [38,39,40]. In this study, YPF was selected as a positive control.

Cellular senescence, a defining feature of aging, is being increasingly identified as a major contributor to chronic lung disease and is frequently linked to impaired function of subcellular organelles [41]. In aged mice, the population of alveolar type 2 (AT2) cells in the lungs is diminished, which is accompanied by an upregulation of inflammation-related genes and a downregulation of genes involved in the cell cycle, ultimately contributing to fibrotic remodeling [42,43]. At present, numerous traditional Chinese medicine formulations and bioactive compounds have demonstrated the capacity to prevent the progression of pulmonary fibrosis by inhibiting the senescence of alveolar type 2 (AT2) cells. Examples include the Mai-wei-yang-fei decoction, resveratrol, and prophylactic quercetin [14,44,45]. These data introduce new avenues for research within our study.

The literature indicates that pulmonary function tests demonstrate changes in lung function in one-year-old mice [1]. In this study, the administration of CT in naturally aged mice significantly increased tidal volume (TV), minute ventilation (MV), peak inspiratory flow (PIF), and peak expiratory flow (PEF), demonstrated a tendency to improve dynamic compliance (Cdyn), and reduced enhanced pause (Penh). These results are consistent with previous reports [46], indicating that CT ameliorates age-related declines in pulmonary function. Moreover, we assessed the expression of senescence-associated proteins p53, p21, and p16, all of which were significantly elevated in aged mice. Similarly, fibrosis markers α-SMA and TGF-β1 were markedly increased in the elderly group, consistent with clinical observations in human lung tissue [47]. Following CT treatment, a significant decrease in both fibrosis and senescence-associated markers was observed in vitro and in vivo. Collectively, these results suggest that CT may have therapeutic potential in mitigating aging-related pulmonary fibrosis and delay aging.

Subsequently, transcriptomic analysis was employed to further investigate the mechanisms. In the differential gene network map are associated with aging, fibrosis, and inflammation, consistent with previous reports [48]. Additionally, upstream regulator analysis identified FBXW7 as a key upstream regulator, and the protein ubiquitination pathway and the cellular response to heat stress were recognized as the principal pathways involved. The existing literature indicates that aging is closely linked to both the protein ubiquitination pathway and the cellular response to heat stress [49]. FBXW7 plays a crucial regulatory role in aging through multiple pathways, including FBXW7/P53 and FBXW7/TPP1. In our data, FBXW7 was decreased in aging mice, but increased in the cellular senescence model. Acute injury and short-term oxidative stress rapidly activate p53, which in turn initiates FBXW7 transcription. During this period, the ubiquitin degradation pathway is not excessively activated. Consequently, both FBXW7 mRNA and protein levels increase [12,13]. Natural aging and long-term chronic aging, as well as prolonged continuous stress, can lead to transcription-protein decoupling, with a compensatory significant increase in FBXW7 gene transcription levels; however, multiple post-transcriptional and post-translational degradation mechanisms overlap, ultimately resulting in a decrease in total FBXW7 protein levels. FBXW7 beta is induced by all tested stress stimuli, primarily but not exclusively in a p53-dependent manner. In contrast, the expression of FBXW7 alpha and gamma isoforms is p53-independent, and their responsiveness to most stress stimuli is limited [50].

Furthermore, it was reported that phosphorylation of S303/S307 residues is required for FBXW7 ubiquitin ligase binding, ubiquitination, and degradation of HSF1 [15]. To elucidate the relationship between FBXW7 and HSF1, we conducted Co-IP experiments using an aging cell model, which confirmed the interaction between FBXW7 and HSF1. HSF1 activity is regulated by its translocation into the nucleus, where it binds to DNA and subsequently activates the expression of downstream targets, including HSP70 and HSP90 [51,52]. Currently, upregulation of heat shock protein 70 expression inhibits fibrosis, mitigates aging, and regulates autophagy [53,54]. In this study, CT markedly increased the expression of nuclear HSF1 protein while concurrently decreasing its cytoplasmic levels. Additionally, CT enhanced the phosphorylation of HSF1 at serine residues 303 and 307 and upregulated the expression of HSP70. Molecular docking analysis further demonstrated that six active compounds can bind to the DNA-binding domain of HSF1. Notably, the active compounds atractylenolide III and tanshinone have been previously reported to possess substantial anti-fibrotic, anti-inflammatory, and anti-aging properties [55,56,57]. These novel findings suggest that CT may attenuate age-related fibrosis potentially through the FBXW7/HSF1/HSP70 pathway, thereby providing protection against protein misfolding stress.

Another critical factor in mitigating the aging process is the enhancement of pulmonary mucosal barrier integrity [47,58]. Cellular senescence or acute lung injury (ALI) in aged mice directly compromises barrier function by downregulating tight-junction proteins such as occludin, ZO-1, and VE-cadherin [58,59]. Furthermore, overexpression of TLR4 may contribute to chronic inflammatory responses [60]. In the present study, administration of CT in naturally aged mice significantly decreased TLR4 expression while substantially increasing the levels of ZO-1, occludin, and claudin 3 proteins. These findings suggest that CT may counteract age-related impairments by promoting the expression of tight-junction proteins.

Focusing on the gut–lung axis has emerged as a promising and practical approach for mitigating respiratory diseases associated with aging [61]. Our experimental results revealed that the CT group exhibited increased abundance of Kineothrix, Vescimonas, and Lawsonibacter, alongside a significant elevation in Flintibacter and Akkermansia. Notably, among these gut microbes, Akkermansia muciniphila (*A. muciniphila*) is associated with aging-related conditions [62]. Administration of AKK PROBIO can increase the concentrations of valeric acid and propionic acid in the intestine, thereby mitigating hepatic fibrosis [63]. Consistent with these findings, our study demonstrated that CT increases the abundance of Akkermansia, slightly elevates propionic acid and valeric acid levels, and enhances the integrity of the intestinal mucosal barrier by restoring mucus secretion from goblet cells, reestablishing continuous MUC2 expression, and upregulating the protein expression of ZO-1, occludin, claudin 3, and claudin 1. Collectively, these results support the role of CT in ameliorating aging and pulmonary fibrosis through modulation of the gut–lung axis.

Certainly, there are several limitations that require further research and improvement. We exclusively used male mice in vivo and only the transformed A549 cell line in vitro, which may introduce bias into the experimental results. Additionally, considering that *C. tangshen* is a medicinal herb used both as food and medicine, its efficacy might be relatively mild. Therefore, a clinical dose eight times higher was selected; however, the overall dosage remains relatively high. Although we performed molecular docking, HSF1 DNA-binding activity in nuclear fractions needs to be detected by EMSA using a radiolabeled HSE oligonucleotide; however, no quantitative analysis of HSF1 binding activity was performed. Additionally, Akkermansia muciniphila (AKK), identified as the most notably affected beneficial bacterium, should be further quantified using RT-PCR. Future studies could investigate its beneficial effects and mechanisms on CT through in vitro culture. Ideally, a co-culture model that simulates an aging cellular environment in conjunction with a pro-fibrotic system ought to be established, enabling gene overexpression or silencing techniques to elucidate the influence of CT on the FBXW7/TPP1 and HSF1/HSP70 signaling pathways.

## 5. Conclusions

The findings of this study indicate that *C. tangshen* may exert anti-aging effects through modulation of the FBXW7–TPP1 axis and potentially mitigate aging-related pulmonary fibrosis via the HSF1/HSP70 signaling pathway. Additionally, it appears to influence the composition of gut microbiota, restore MUC2 expression, and improve colonic barrier integrity. Collectively, these results suggest that Codonopsis tangshen Oliv. represents a promising botanical candidate for the treatment of aging and related fibrotic lung injury. Nonetheless, further mechanistic investigations and translational research are necessary to validate these preliminary findings.

## Figures and Tables

**Figure 1:**
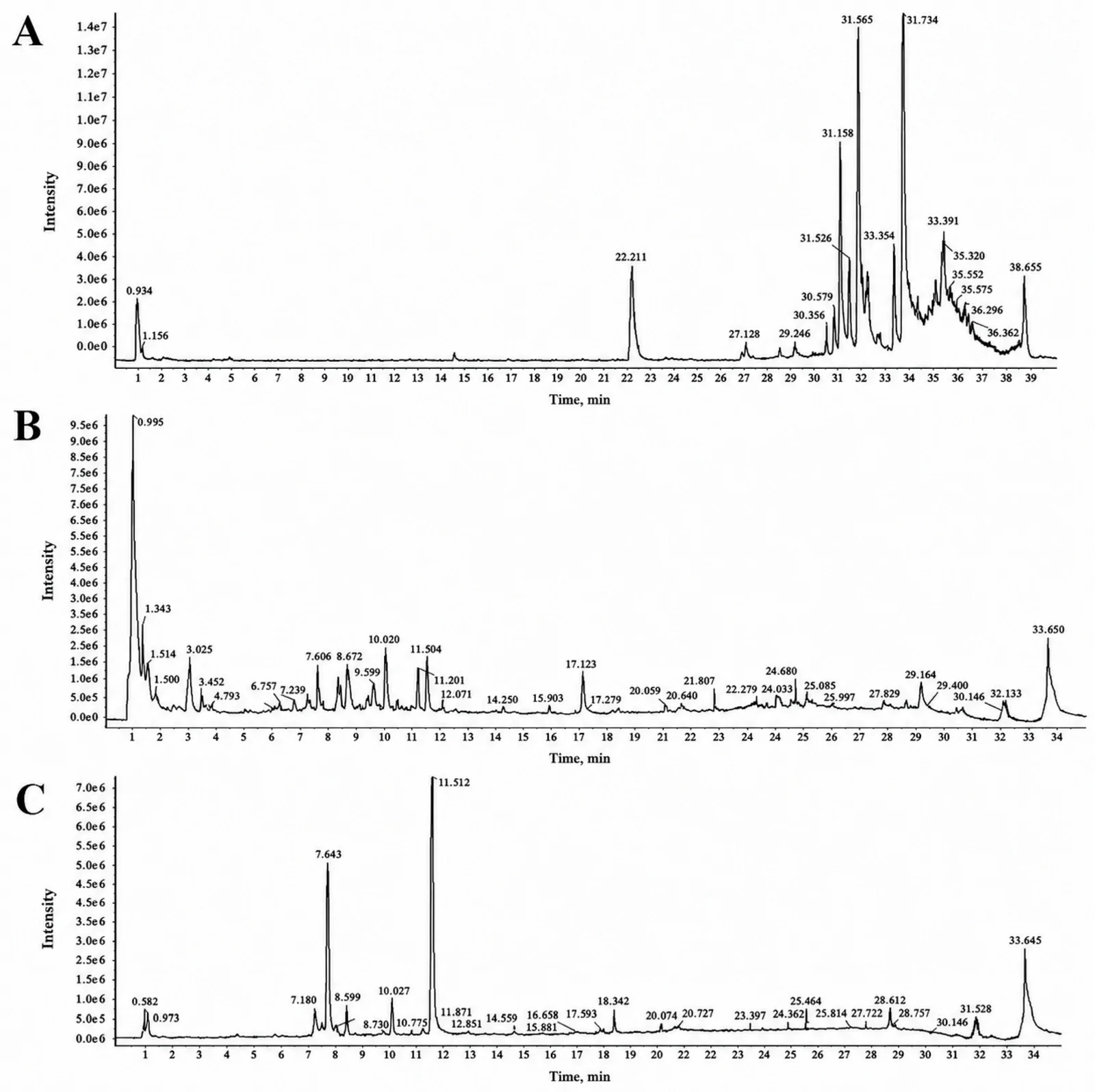
Total ion chromatograms (TIC) of *C. tangshen* and the mixed standard ((**A**) positive; (**B**) negative; (**C**) mixed standard of Codonopsis glycoside I and lobetyolin).

**Figure 2:**
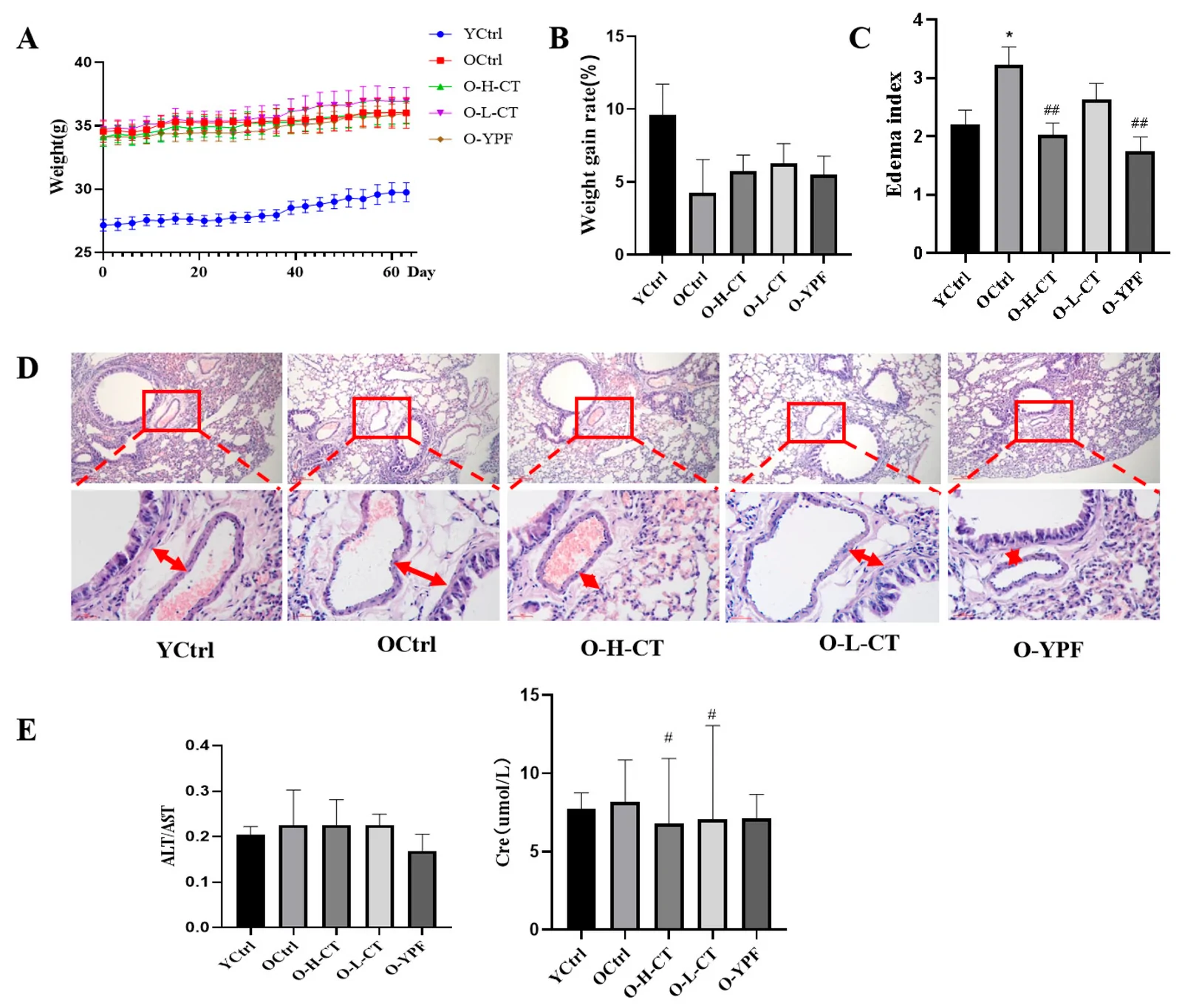
Effects of *C. tangshen* on body weight, senescence-associated β-galactosidase (SA-β-gal) staining, edema index, and fibrosis, as evaluated by Masson staining, in aged mice. (**A**) Body weight. (**B**) Body weight gain rate. (**C**) Edema index. (**D**) Hematoxylin and eosin (H&E) staining. (**E**) Liver and kidney function. Compared to YCtrl * *p* < 0.05; compared to OCtrl. ^#^ *p* < 0.05, ^##^ *p* < 0.01. Data are expressed as means ± SD (*n* = 6).

**Figure 3:**
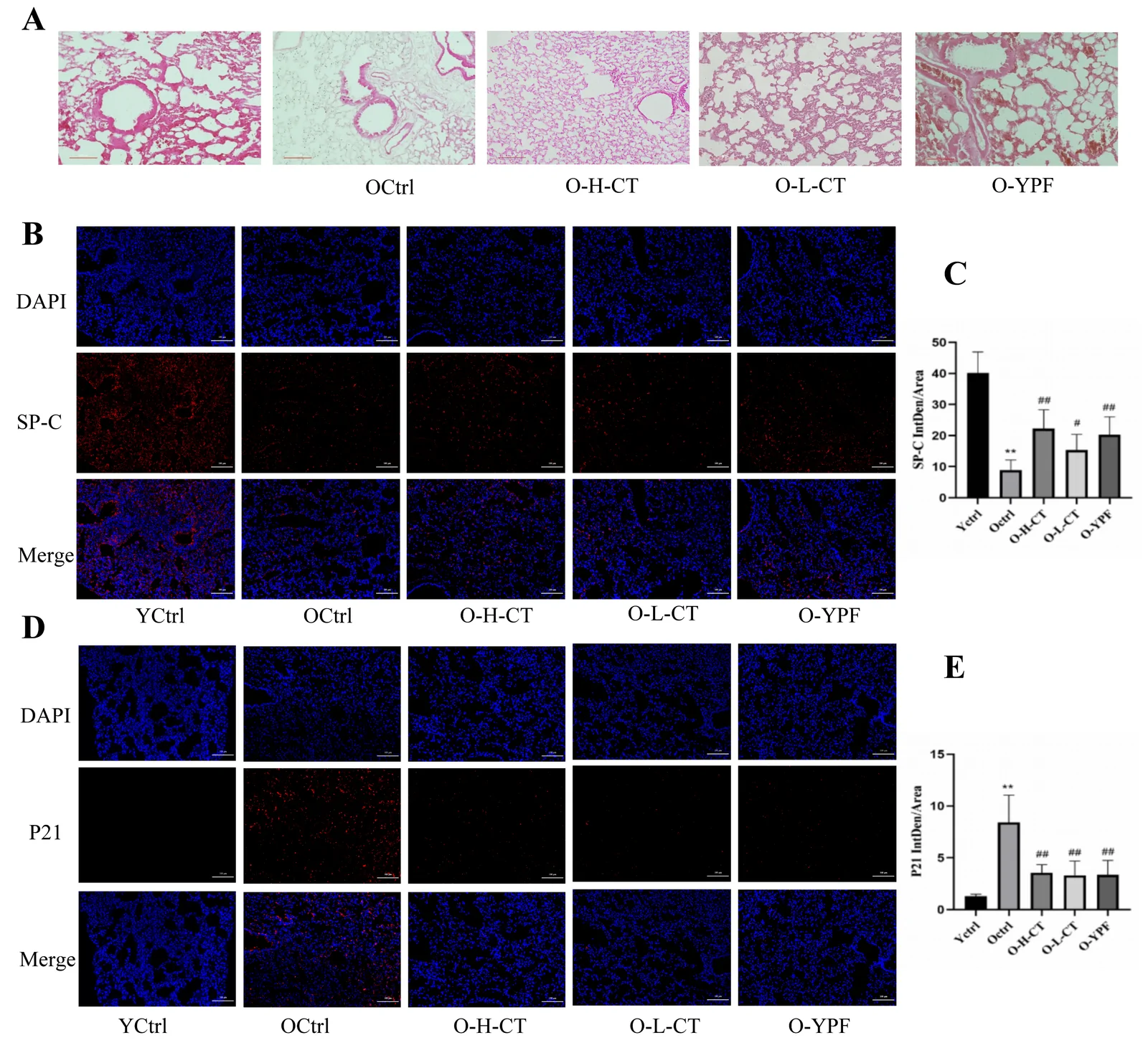
Aging-associated SA-β-gal staining and IF analysis of SP-C and P21. (**A**) Aging-associated SA-β-gal staining. (**B**,**C**) IF staining of SP-C and analysis. (**D**,**E**) IF staining of P21 and analysis. Data are expressed as means ± SD (*n* = 3). ** *p* < 0.01 vs. YCtrl group; ^#^ *p* < 0.05 and ^##^ *p* < 0.01 vs. OCtrl group.

**Figure 4:**
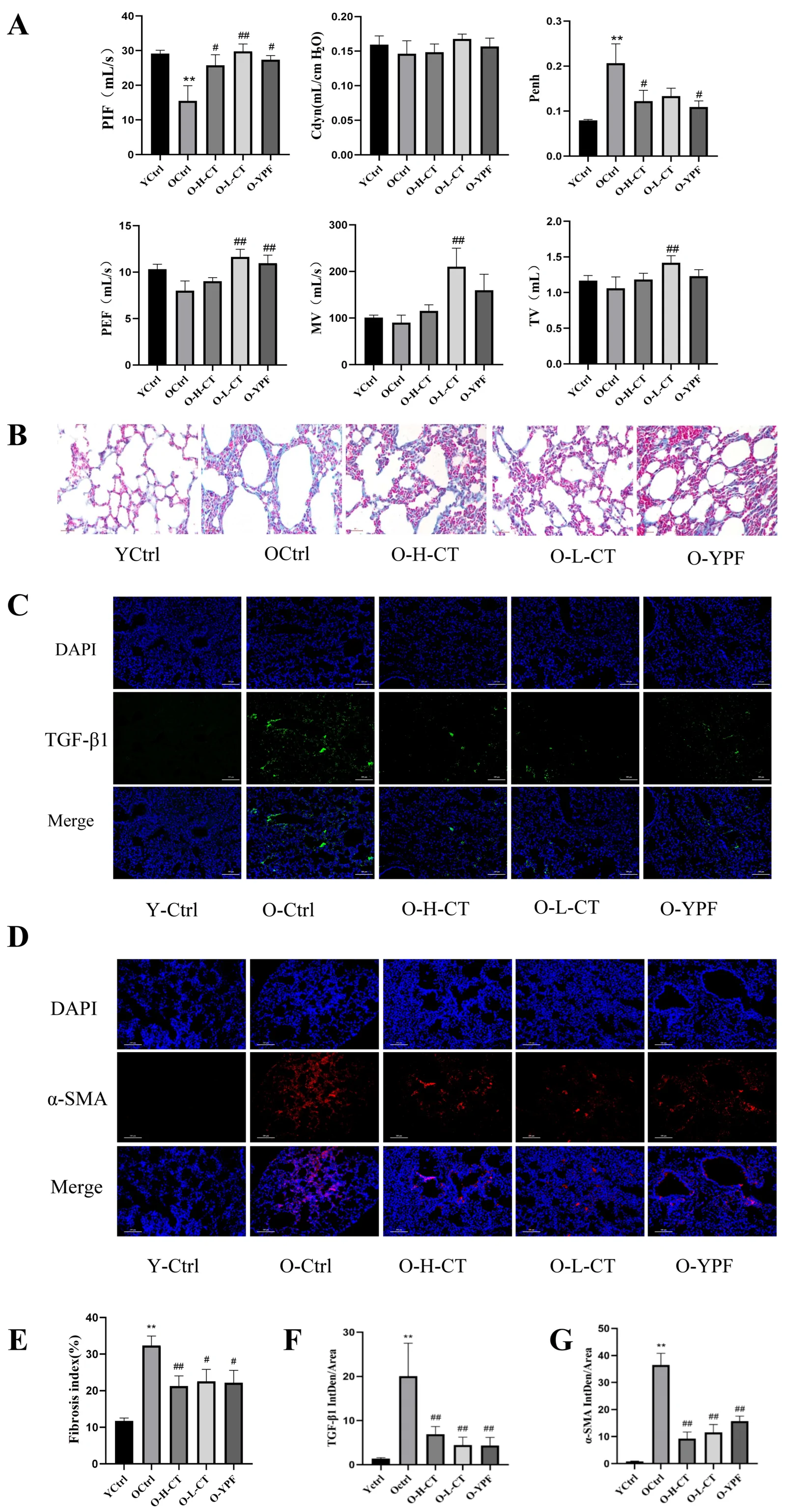
Effects of CT on pulmonary ventilation parameters, Masson staining and IF analysis of α-SMA and TGF-β1 in an aged murine model. (**A**) Pulmonary ventilation parameters, *n* = 6. (**B**) Masson staining. (**C**,**F**) IF analysis of TGF-β1. (**E**) Analysis of the Fibrosis Index from Masson’s Staining. (**D**,**G**) IF analysis of α-SMA. Data are expressed as means ± SD (*n* = 3). ** *p* < 0.01 vs. YCtrl group; ^#^ *p* < 0.05 and ^##^ *p* < 0.01 vs. OCtrl group.

**Figure 5:**
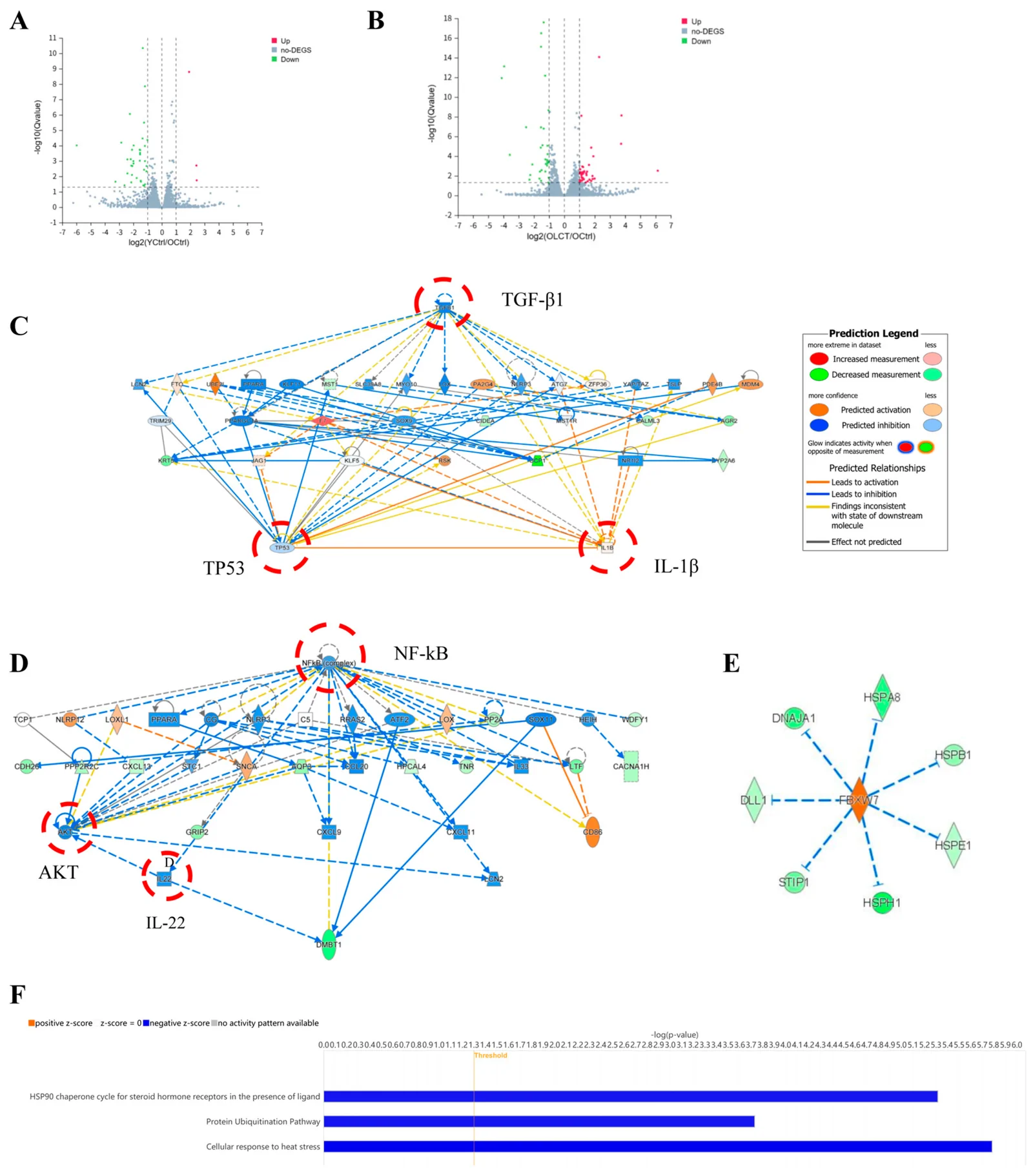
Differential gene expression and IPA network analysis between YCtrl and OCtrl Groups. (**A**) Volcano plot showing differentially expressed genes in lung tissue comparing YCtrl and OCtrl groups. (**B**) Volcano plot of differentially expressed genes in lung tissue comparing OCtrl and CT treatment. (**C**,**D**) Network analysis of differential genes. (**E**) Upstream regulator analysis. (**F**) Canonical pathway analysis.

**Figure 6:**
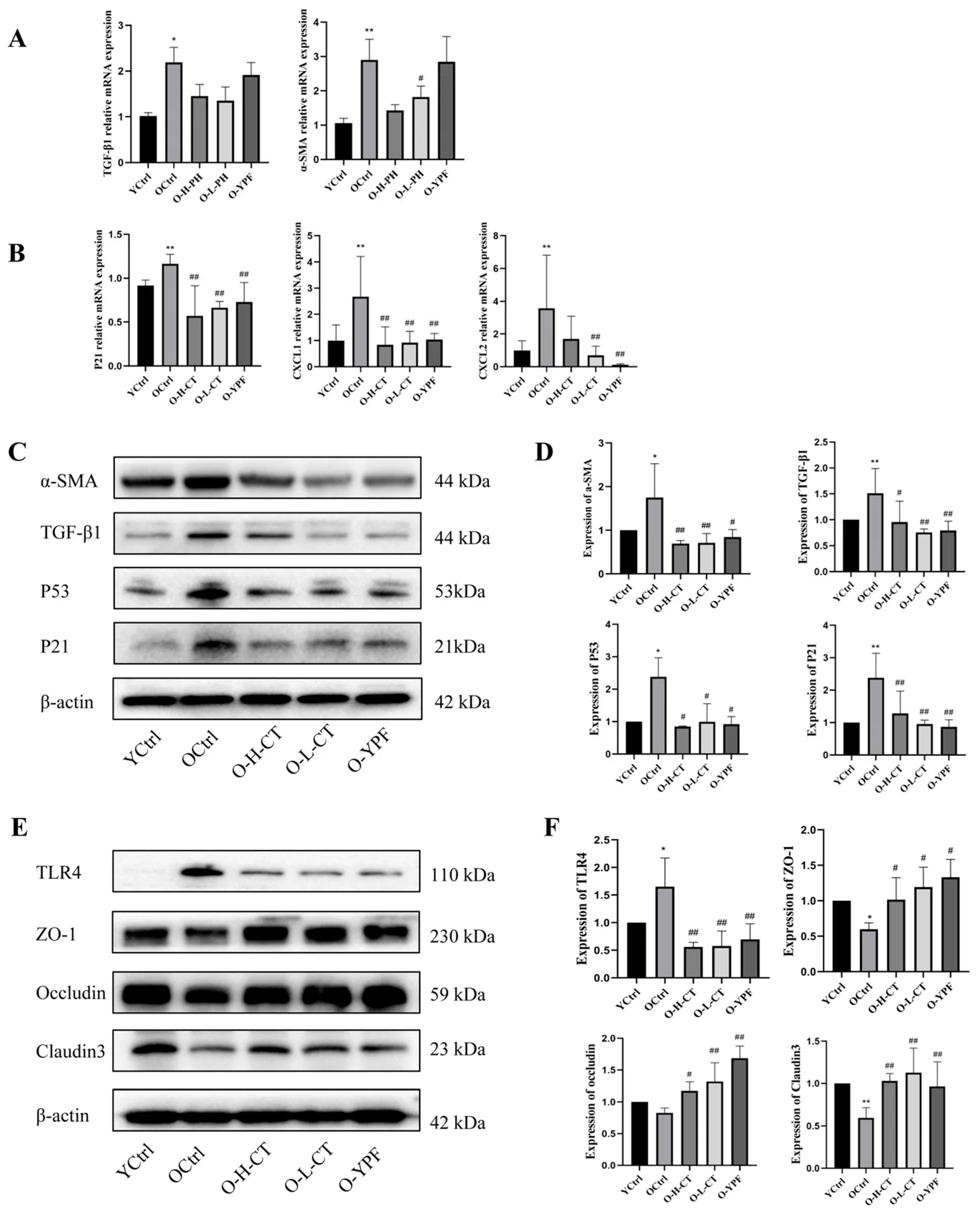
Effects of *C. tangshen* on fibrosis, SASP-related markers, and pulmonary mucosal barrier in aging mice. (**A**) Fibrosis-related gene expression. (**B**) SASP-related gene expression. (**C**,**D**) Protein expression on fibrosis, SASP-related markers, and analysis. (**E**,**F**). Key protein expression on the pulmonary mucosal barrier and analysis. Data are expressed as means ± SD (*n* = 3). * *p* < 0.05 and ** *p* < 0.01 vs. YCtrl group; ^#^ *p* < 0.05 and ^##^ *p* < 0.01 vs. OCtrl group.

**Figure 7:**
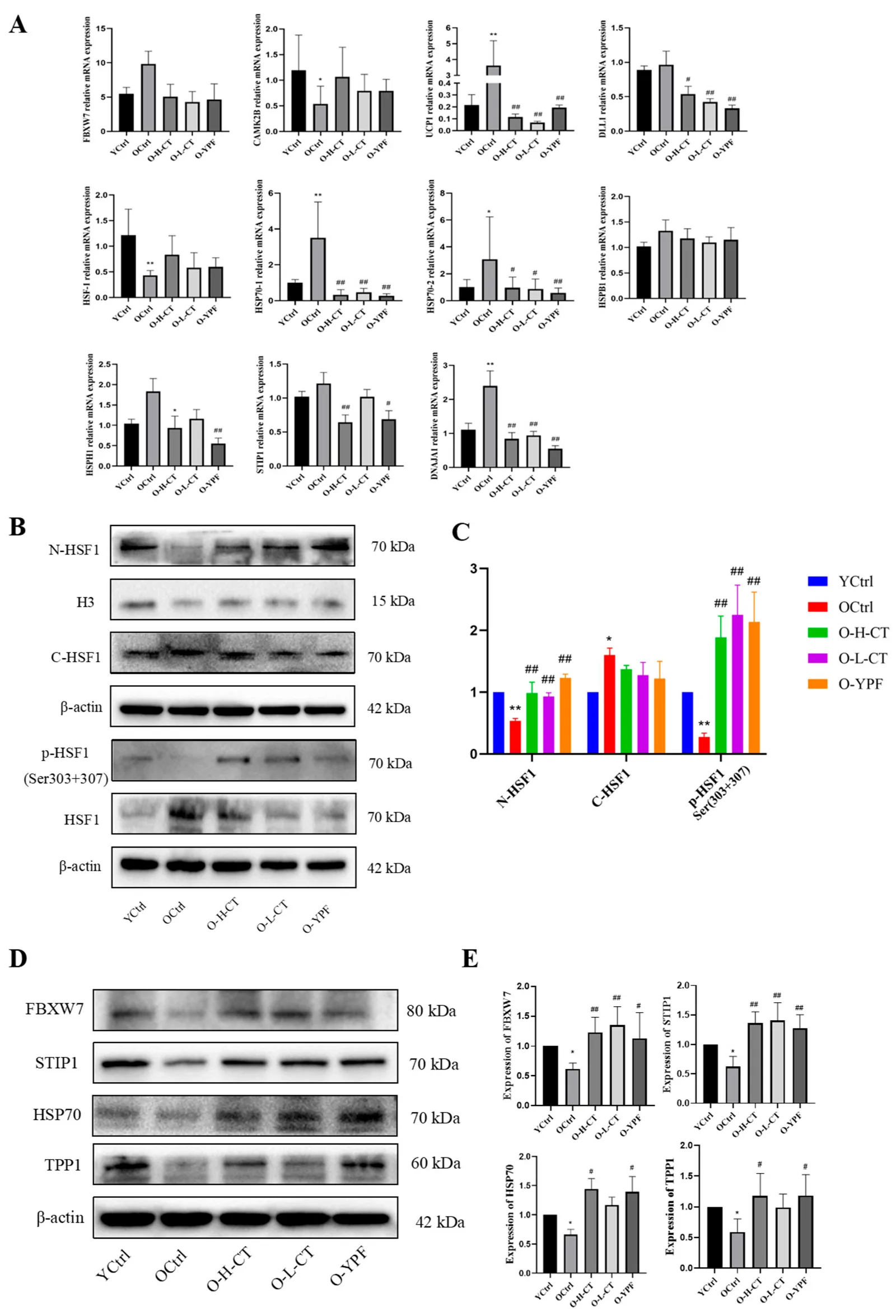
*C. tangshen* effects on the FBXW7/TPP1 and HSF1/HSP70 pathway in aged pulmonary tissue. (**A**) RT-qPCR analysis of heat shock-related genes. (**B**,**C**) Nuclear, cytoplasmic, and phosphorylated expression of HSF1 in aged pulmonary tissue. (**D**) Western blot analysis of FBXW7/TPP1 and HSP70. (**E**) Expression levels of related proteins. Data are expressed as means ± SD (*n* = 3). * *p* < 0.05 and ** *p* < 0.01 vs. YCtrl group; ^#^ *p* < 0.05 and ^##^ *p* < 0.01 vs. OCtrl group.

**Figure 8:**
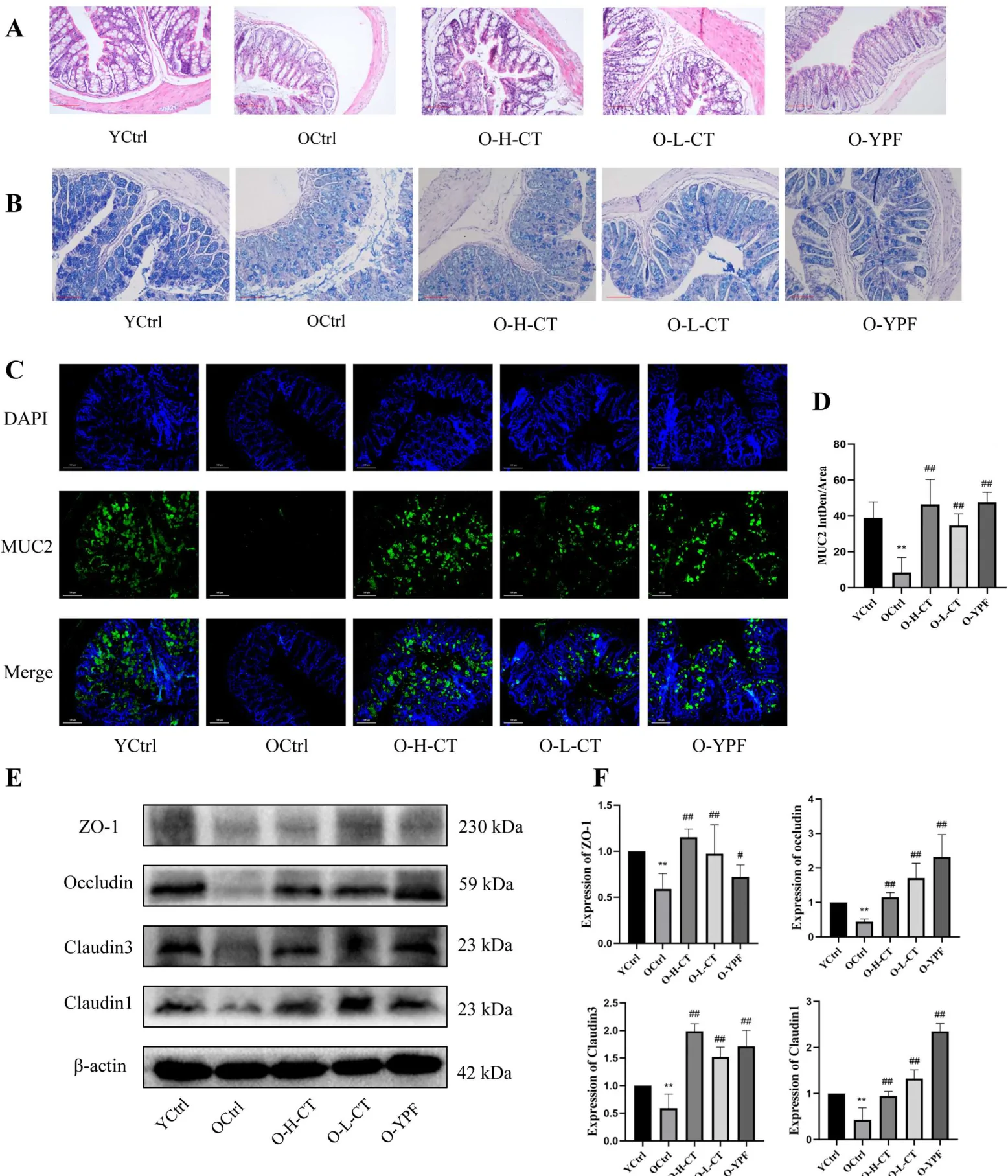
Effects of *C. tangshen* on intestinal barrier function and related proteins in aged colon tissue. (**A**) HE staining. (**B**) AB-PAS staining. (**C**) IF staining of MUC2. (**D**) Analysis of MUC2 by ImageJ. (**E**) Western blot analysis of tight-junction protein expression in the colon. (**F**) Statistical analysis of relative protein expression levels. Data are expressed as means ± SD (*n* = 3). ** *p* < 0.01 vs. YCtrl group; ^#^ *p* < 0.05 and ^##^ *p* < 0.01 vs. OCtrl group.

**Figure 9:**
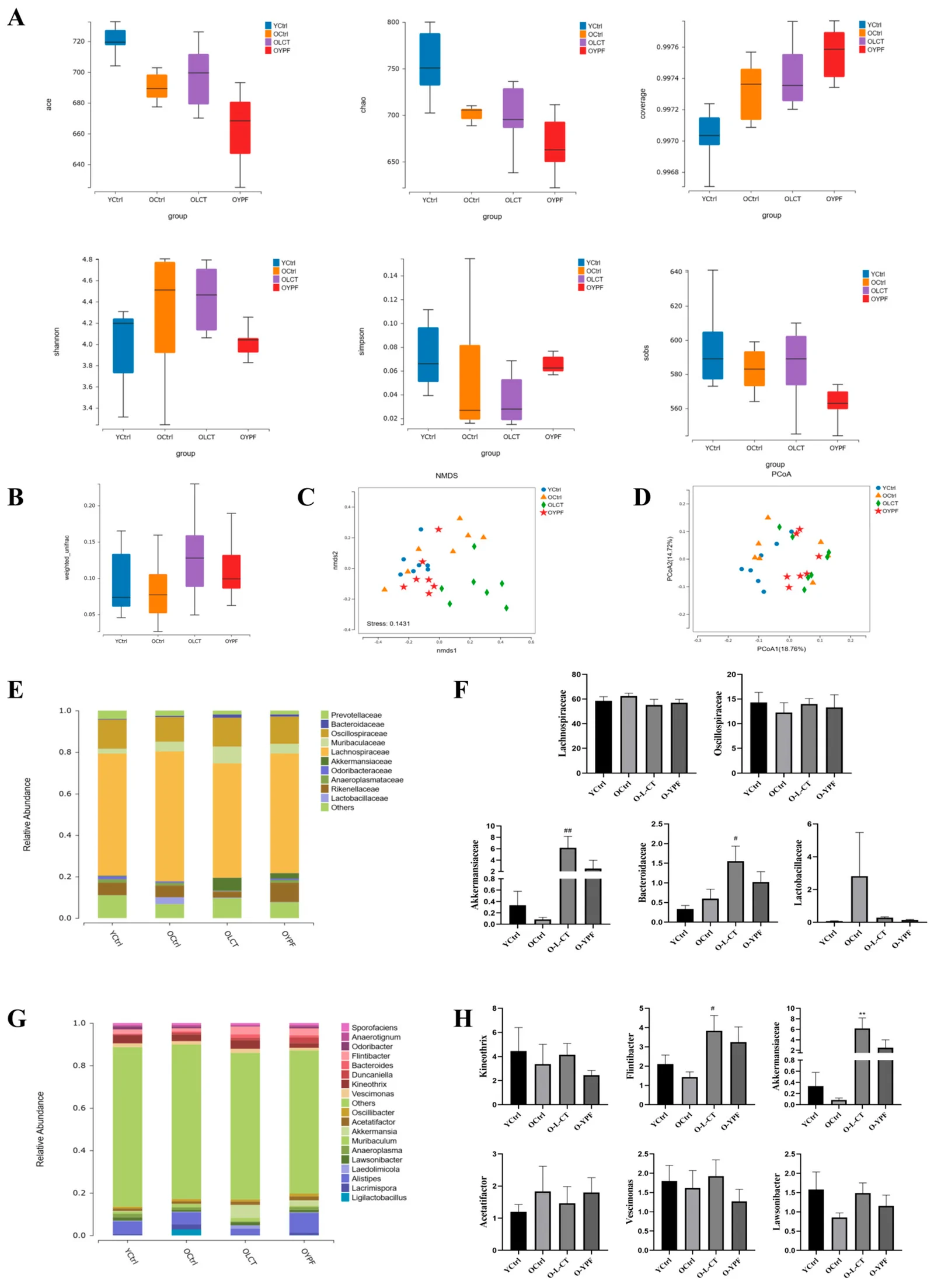
Effects of *C. tangshen* on gut microbiota in aged mice. (**A**) α-diversity analysis. (**B**) β-diversity analysis. (**C**) NMDS analysis. (**D**) PCoA analysis. (**E**,**F**) The ten most predominant and significantly different gut microbiota at the family level. (**G**,**H**) The ten most predominant and significantly different gut microbiota at the genus level. Data are expressed as means ± SD (*n* = 6). ** *p* < 0.01 vs. YCtrl group; ^#^ *p* < 0.05 and ^##^ *p* < 0.01 vs. OCtrl group.

**Figure 10:**
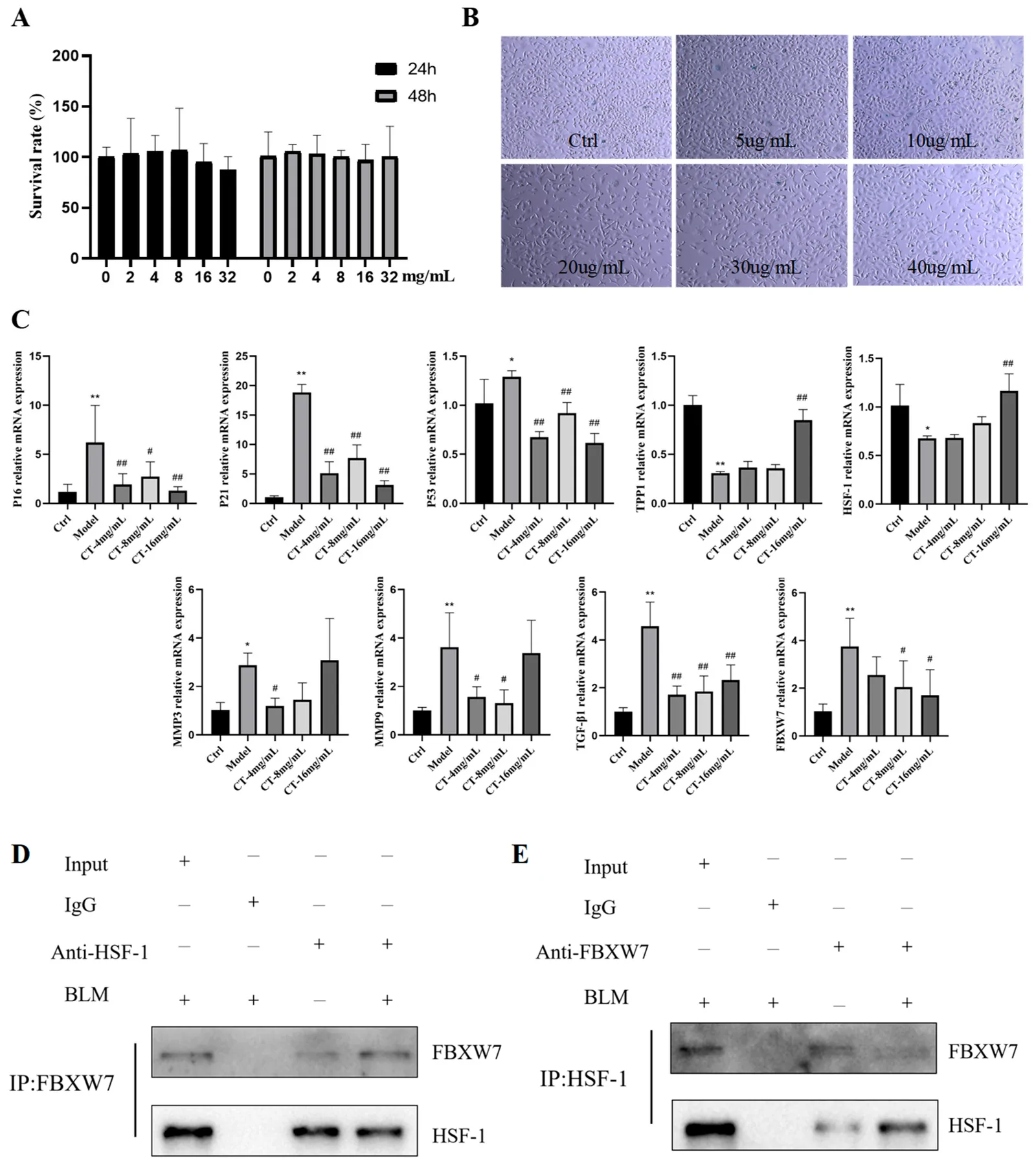
Effects of *C. tangshen* on cell senescence model and Co-IP analysis. (**A**) Survival rate of A549 cells after treatment with CT. (**B**) Different concentrations of bleomycin-induced cellular senescence models based on SA-β-gal staining. (**C**) The effect of CT on gene expression levels was evaluated using RT-qPCR. (**D**,**E**) Co-IP analysis of the interaction between FBXW7 and HSF1. Data are expressed as means ± SD (*n* = 3). * *p* < 0.05 and ** *p* < 0.01 vs. YCtrl group; ^#^ *p* < 0.05 and ^##^ *p* < 0.01 vs. OCtrl group.

**Figure 11:**
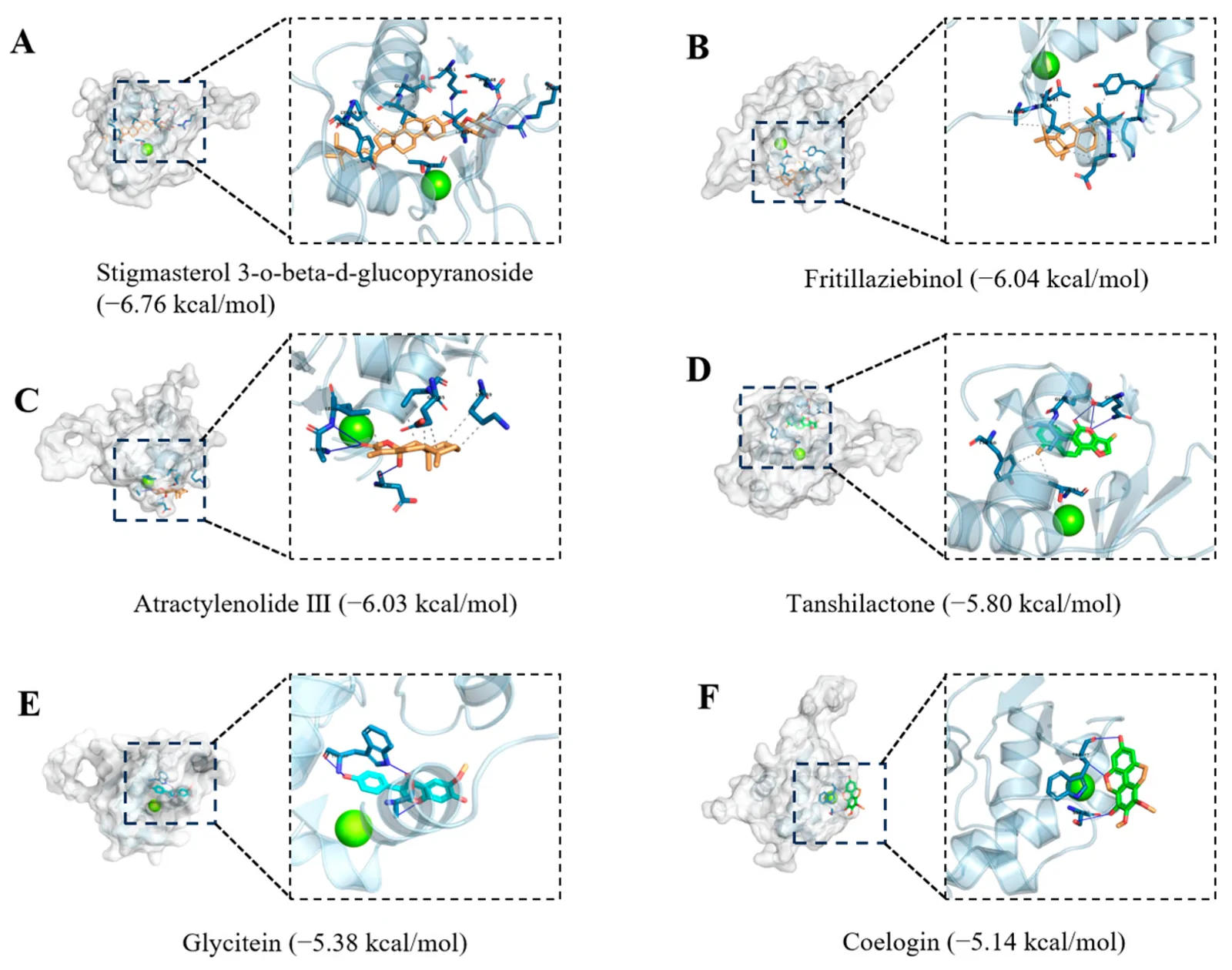
Visualization in *C. tangshen* component docking with key target HSF1 molecules. The protein backbone was presented in cartoon style (cyan), while the binding pocket was displayed as a transparent gray surface. The metal cofactor within the pocket was marked as a green sphere. Ligands were represented by stick models, and interacting amino acid residues were colored in blue. White dotted lines represented hydrogen-bond interactions between ligands and protein residues. (**A**) 3-O-beta-D-glucopyranoside acts on HSF1. (**B**) Fritillaziebinol acts on HSF1. (**C**) Atractylenolide III acts on HSF1. (**D**) Tanshilactone acts on HSF1. (**E**) Glycitein acts on HSF1. (**F**) Coelogin acts on HSF1.

**Table 1:** Identification of active components in *Codonopsis tangshen* Oliv. aqueous extracts using UHPLC-Q-TOF/MS.

Number	Ion Mode	Retention Time	Molecular Formula	Theoretical Relative Molecular Mass	Measured Relative Molecular Mass	Error PPM	Secondary Fragment	Name
1	M−	0.98	C_25_H_24_O_12_	191.0561	191.0559	−1.1	127.0438, 173.0460, 191.0571	Quinic Acid
2	M−	1.06	C_4_H_6_O_5_	133.0142	133.0153	7.9	115.0061, 71.0215	Malic Acid
3	M+	1.26	C_14_H_21_NO_5_	284.1492	284.1492	0.0	88.0766, 118.0860, 137.0574	Codonopsinol
4	M+	1.38	C_5_H_5_N_5_	136.06232	136.06183	−3.6	119.0352, 92.0250, 136.0620	Adenine
5	M−	1.43	C_5_H_11_NO_2_	118.0862	118.0868	4.6	72.0834, 58.0676	Betaine
6	M−	1.57	C_7_H_6_O_3_	137.0244	137.0249	3.5	93.0387	3-Hydroxybenzoic acid
7	M+	1.60	C_12_H_17_NO_4_	240.1230	240.1227	−1.4	74.0622, 107.0505	Codonopsinol C
8	M−	1.79	C_7_H_6_O_3_	137.0244	137.0255	7.9	93.0382	3,4-Dihydroxybenzaldehyde
9	M+	1.84	C_9_H_11_N_3_	182.08172	182.08145	−1.5	136.0757, 147.0441, 165.0546	Tyrosine
10	M+	1.99	C_10_H_13_N_5_O_4_	268.10458	268.10449	−0.3	136.061	Adenosine
11	M+	2.80	C_9_H_11_NO_2_	166.0868	166.086	−4.8	120.0805, 103.0545	Phenylalanine
12	M+	3.05	C_19_H_29_NO_9_	416.19206	416.1894	−6.4	266.1234, 254.1387, 416.1894	Codonopiloside A
13	M+	3.36	C_14_H_21_NO_4_	268.1543	268.1536	−2.7	88.0763, 118.0862, 121.0648	Codonopyrrolidium B
14	M+	3.49	C_14_H_21_NO_4_	268.1543	268.1541	−0.9	88.0765, 268.1541	Codonopsine
15	M+	3.68	C_13_H_19_NO_5_	270.1336	270.1339	1.1	74.0603, 270.1516	Codonopsinol A
16	M+	3.69	C_13_H_19_NO_3_	238.14432	238.1438	−2.2	121.0648, 238.1438	Codonopsinol B
17	M+	3.69	C_13_H_19_NO_3_	238.14432	238.1438	−2.2	202.1332, 121.0648, 238.14380	Codonopsinine
18	M+	4.5	C_11_H_12_N_2_O_2_	205.0977	205.09678	−4.5	188.0706, 170.0600, 159.0917	Tryptophan
19	M−	4.94	C_16_H_18_O_9_	353.0878	353.0857	−6.0	191.0562	5-Caffeoylquinic acid
20	M−	4.97	C_16_H_18_O_9_	353.0878	353.08590	−5.4	191.0562	Chlorogenic acid
21	M−	5.04	C_21_H_20_O_10_	431.0983	431.0961	−5.3	135.0461, 283.0600, 311.0560	apigenin 7-O-glucoside
22	M−	5.29	C_16_H_18_O_9_	353.0878	353.0860	−5.1	191.0588, 173.0445, 135.0432, 93.0368	Cryptochlorogenic acid
23	M−	5.31	C_15_H_18_O_8_	325.09234	325.0921	−0.7	163.0439, 205.0534	4′-O-beta-D-glucosyl-cis-p-coumaric acid
24	M+	5.52	C_7_H_6_O_3_	139.03952	139.03862	−6.5	95.0498, 77.0387, 121.0304	4-Hydroxybenzoic acid
25	M−	5.60	C_17_H_24_O_10_	387.1296	387.1270	−6.9	387.1431, 207.0659, 143.0391	Geniposide
26	M−	5.64	C_9_H_8_O_4_	179.03443	179.03554	6.2	135.0477	Caffeic acid
27	M−	6.78	C_18_H_32_O_11_	423.18664	423.18414	−5.9	261.1325, 221.0647, 179.0550, 161.0459,	Trans-2-hydroxy-β-sophoroside
28	M+NH4	7.1	C_17_H_24_O_9_	390.17641	390.1767	0.7	211.0966, 193.0860, 161.0588	Syringin
29	M−	7.23	C_18_H_32_O_11_	423.18664	423.18418	−6.1	261.1344, 221.0644, 179.0578, 161.0479,	Cis-2-hydroxy-β-sophoroside
30	M−	7.57	C_29_H_42_O_18_	677.22929	677.22528	−5.9	497.1644, 453.1741	Tangshenoside I
31	M−	8.37	C_18_H_34_O_11_	425.20229	425.2058	8.3	263.1482, 149.0457	Hexyl beta-sophoroside
32	M−	9.41	C_17_H_32_O_10_	395.19172	395.1896	−5.4	263.1504, 101.0270	Pentose-6-O-hexyl glucoside
33	M−-	9.68	C_26_H_38_O_13_	557.2239	557.2197	−7.7	89.0276, 143.0740, 305.1231	Lobetyolinin isomer
34	M−	10.61	C_9_H_16_O_4_	187.09703	187.098	5.2	169.0875, 187.0980	Azelaic acid
35	M+FA-	10.81	C_20_H_30_O_8_	443.1922	443.1890	−7.4	89.0388, 119.0393, 217.1258	Cordifolioidyne B
36	M−	10.9	C_10_H_10_O_4_	193.05008	193.05077	3.6	161.0193, 133.0314	Ferulic acid
37	M−	11.17	C_26_H_38_O_13_	557.2239	557.2207	−5.9	119.0486, 161.0476, 323.0979	Lobetyolinin
38	M−	11.48	C_20_H_28_O_8_	395.17059	395.16875	−4.7	233.1127, 215.1084	Lobetyolin
39	M−	11.76	C_38_H_48_O_20_	823.26607	823.26009	−7.3	497.1637, 453.1736, 325.0924, 163.0414	6‴-Trans-p-coumaroyl tangshenoside I
40	M−	12.08	C_38_H_48_O_20_	823.26607	823.2647	−1.7	495.1642, 453.1729, 325.0907, 163.0403	6‴-Cis-p-coumaroyl tangshenoside I
41	M−	12.12	C_15_H_10_O_4_	253.0506	253.0501	−2.1	223.0466, 135.0036	chrysin
42	M+	12.36	C_19_H_28_NO_6_	367.1945	367.1947	0.2	83.0511, 137.0590, 170.1160	3′-Hydroxy codonopyrrolidium C
43	M−	12.96	C_15_H_10_O_6_	285.03991	285.0396	−1.1	133.0295, 151.0037, 285.0396	Luteolin
44	M−	13.46	C_10_H_10_O_3_	177.0557	177.0557	0.0	117.0368, 145.0308	Coniferaldehyde
45	M−	13.92	C_21_H_26_O_12_	469.1346	469.1362	3.4	325.0929, 163.040, 119.0502	Tangshenoside V Isomer
46	M−	13.97	C_21_H_26_O_12_	469.1346	469.1337	−1.9	325.0926, 163.0409, 119.0523	Tangshenoside V
47	M+	14.16	C_19_H_27_NO_5_	350.1962	350.1961	−0.3	83.0509, 187.0756, 219.1253	Codonopyrrolidium C
48	M+	14.36	C_19_H_28_NO_5_	351.19675	351.1981	3.8	250.1438, 220.1332	Codonopyrrolidium A
49	M−	15.26	C_16_H_12_O_6_	299.0561	299.0552	−3.0	283.0312, 256.0382, 227.0339	Chrysoeriol
50	M−	15.32	C_16_H_12_O_6_	299.0561	299.0547	−4.7	255.0295, 283.0211, 299.0553	Diosmetin
51	M−	15.93	C_18_H_32_O_5_	327.21715	327.217	−0.5	211.1340, 229.1440	9,12,13-Trihydroxy-10,15-octadecadienoic acid
52	M+	16.61	C_15_H_22_O	219.1743	219.1745	0.7	135.0812, 203.1452, 219.1740	Zerumbone
53	M−	16.79	C_18_H_34_O_5_	329.2328	329.23221	−1.8	211.1309, 229.1386	9,12,13-Trihydroxy-10-octadecenoic acid
54	M−	20.12	C_17_H_26_O_4_	293.1748	293.1758	−3.5	177.0934, 211.1544	6-gingerol
55	M+H−H_2_O	21.18	C_14_H_18_O_3_	217.1223	217.1226	1.3	199.1099, 127.0817, 91.0568	Lobeyol isomer
56	M−	21.85	C_18_H_34_O_4_	313.23788	313.23773	−0.5	201.1131, 313.2356	9,10-Dihydroxy-12-octadecenoic acid
57	M−	24.13	C_18_H_32_O_3_	295.22732	295.22687	−1.5	277.2165, 295.2277, 171.1040	9-Hydroxy-10,12-octadecadienoic acid
58	M+H−H_2_O	24.71	C_14_H_18_O_3_	217.1223	217.1221	−1.0	199.1099, 91.0548	Lobeyol
59	M−	26.65	C_16_H_32_O_2_	255.2329	255.2321	−3.3	255.2308, 201.8358	Palmitic acid
60	M−	28.81	C_37_H_46_O_12_	681.2916	681.2917	0.1	78.9634, 681.2911	Agrimol B
61	M−	32.31	C_18_H_36_O_2_	283.2642	283.2634	−3.0	283.2630	Stearic acid
62	M+	32.33	C_15_H_20_O_3_	249.1485	249.1474	−4.5	69.0697, 107.0871, 203.1466, 231.1344	Atractylenolide III
63	M+	34.26	C_15_H_20_O_2_	233.1536	233.1521	−6.5	107.0824, 187.1475, 215.1431, 233.1588	Atractylenolide II
64	M+	35.60	C_15_H_18_O_2_	231.1379	231.1375	−2.0	185.1322, 203.1113, 231.1398	Atractylenolide I
65	M+	37.32	C_30_H_48_O_3_	457.3676	457.3664	−2.7	421.3578, 439.3517, 457.3608	Ursolic acid
66	M+	37.54	C_30_H_48_O_4_	473.3625	473.3618	−1.6	217.1229, 437.3486, 455.3428, 473.3391	Choushenpilosulyne C

**Table 2:** Reference substance degradation information.

Serial Number	Ion Pattern	Retention Time	Molecular Formula	Theoretical Molecular Weight	Measured Molecular Weight	Error (PPM)	Secondary Fragment	Compound	Type
1	M−	7.67	C_29_H_42_O_18_	677.2293	677.2262	−4.6	497.1650, 261.0974, 161.0477	Codonopsis Glycoside I	Phenylpropanoids
2	M−	11.46	C_20_H_28_O_8_	395.1706	395.16790	−6.8	143.0731, 159.0823, 215.1100	Lobetyolin	Alkyne

## Data Availability

The original contributions presented in the study are included in the article/Supplementary Materials. Further inquiries can be directed to the corresponding author.

## Supplementary Materials

The following supporting information can be downloaded at https://www.mdpi.com/article/10.3390/nu18193289/s1. Table S1: RT-qPCR primer sequences for mouse and human.
