# Supplementary material for: Codonopsis tangshen Attenuates Age-Related Pulmonary Fibrosis Through Senescence and Gut–Lung Axis Modulation
Source: Nutrients. 2026 Oct 7;18(19):3289. doi: 10.3390/nu18193289 (PMC13667169; doi:10.3390/nu18193289)
Supplement: Supplementary file 1 [file nutrients-18-03289-s001.zip › nutrients-4508119-supplementary.pdf]

Table S1 RT-qPCR primer sequences for mouse and human

Species	Gene name	Gene sequence number	Upstream and downstream primers	Product length
Mouse	*β-actin*	NM_007393.5	CCTCACTGTCCACCTTCCA<br>CCATGCCAATGTTGTCTCTTAT	295 bp
	Tgf-β1	NM_011577.2	GGATACCAACTATTGCTTCAG<br>TGTCCAGGCTCCAAATATAG	147 bp
	α-sma	NM_007392.4	TTTGCTGACCTCTCTGAGGC<br>CTCCAGGGACTCGTTAGTGC	107 bp
	Il-17	NM_010552.3	CCTGGACTCTCCACCGCAA<br>TTCCCTCCGCATTGACACAG	110 bp
	Il-6	NM_031168.2	TAGTCCTTCCTACCCCAATTTCC<br>TTGGTCCTTAGCCACTCCTTC	101 bp
	Ifn-γ	NM_008337.4	AGACAATCAGGCCATCAGCAA<br>GTGGGTTGTTGACCTCAAACT	114 bp
	Il-1β	NM_008361.4	TGCCACCTTTTGACAGTGATG<br>TGATGTGCTGCTGCGAGATT	100 bp
	Il-22	NM_015747.3	TCGCCTTGATCTCTCCACTC<br>GCTCAGCTCCTGTCACATCA	131 bp
	P21	NM_007669.5	CCTGGTGATGTCCGACCTG<br>CCATGAGCGCATCGCAATC	109 bp
	Cxcl1	NM_008176.3	CTGGGATTCACCTCAAGAACATC<br>CAGGGTCAAGGCAAGCCTC	103 bp
	Cxcl2	NM_009140.3	CCAACCACCAGGCTACAGG<br>GCGTCACACTCAAGCTCTG	102 bp
	Mmp3	NM_010809.3	TGGAGATGCTCACTTTGACG<br>GAGAGATGGAAACGGGACAA	115 bp
	Mmp9	NM_013599.5	CTACATAGACGGCATCCAG<br>CTGTCGGCTGTGGTTCAGT	117 bp
	Hsf-1	NM_008295.3	ATCTCACTGGTGCAGTCGAAC	119 bp

Species	Gene name	Gene sequence number	Upstream and downstream primers	Product length
	Hsp70-1	NM_010478.3	TGGAGATGGAGCTGAGTATGG<br>GGTGCTGACGAAGATGAAGGAGATC<br>CTGCCGCTGAGAGTCGTTGAAG	127 bp
	Hsp70-2	NM_010479.3	ACAAGTCGGAGAACGTGCAGGA<br>GTTGTCCGAGTAGGTGGTGAAG	121 bp
	Hspb1	NM_013560.3	GCTCACAGTGAAGACCAAGGAAGG<br>GCACCGAGAGATGTAGCCATGTTC	116 bp
	Hsph1	NM_008305.3	CGCAGATTGTGGGCTTGA<br>TGGCTTCTCCTCGGCATT	105 bp
	Stip1	NM_019716.3	TGAGTGCTGGGAACATTGATG<br>AGTCTCCTTTCTTGGCGTAGG	111 bp
	Dnaja1	NM_007850.3	CCTAAGGAAAAGCTCAAAGA<br>AAAGTGGATAAGCCAAGAC	124 bp
	FBWX7	NM_001428748.1	GACGCCGAATTACATCTGTC<br>GTAGCAGGTCTTTGGGTTC	243 bp
Human	*Gapdh*	NM_002046.7	ATGGGGAAGGTGAAGGTCGGAGT<br>TAGTTGAGGTCAATGAAGGGGTC	179 bp
	P16	NM_000077.5	GCTTCCTGGACACGCTGGTGGT<br>GGCATCTATGCGGGCATGGTTA	151 bp
	P21	NM_000389.5	CCCGTGAGCGATGGAACT<br>CGAGGCACAAGGGTACAAG	157 bp
	P53	NM_000546.6	CAGCACATGACGGAGGTTGT<br>TCATCCAAATACTCCACACGC	141 bp
	Tpp1	NM_000391.4	CTAATTCTGAGCTCTCTAGGCC<br>TATGAGGGTCAGAGATAGGTCC	202 bp
	Hsf-1	NM_005526.4	GCCAGTTTGCCAAGGAGGT<br>TGTCGTCTCTCTCTGGCTTGAC	174 bp

Species	Gene name	Gene sequence number	Upstream and downstream primers	Product length
	Mmp3	NM_002422.5	CACTCACAGACCTGACTCGGTT<br>AAGCAGGATCACAGTTGGCTGG	231 bp
	Mmp9	NM_004994.3	TCTGGAGGTTCGACGTGAA<br>AGAAATAGGCTTTCTCTCGGTACT	203 bp
	Tgf-β1	NM_000660.7	CGACTCGCCAGAGTGGTTAT<br>TAGTGAACCCGTTGATGTCCA	224 bp
	FBWX7	NM_033632.3	TGCAAAGTCTCAGATTATACC<br>ACTTCTCTGGTCCGCTCCAGC	187 bp
